# Considerations on the Controlled Delivery of Bioactive Compounds through Hyaluronic Acid Membrane

**DOI:** 10.3390/membranes12030303

**Published:** 2022-03-08

**Authors:** Eugenia Eftimie Totu, Daniela Mănuc, Tiberiu Totu, Corina Marilena Cristache, Roxana-Mădălina Buga, Fatih Erci, Camelia Cristea, Ibrahim Isildak

**Affiliations:** 1 Department of Analytical Chemistry and Environmental Engineering, University Politehnica of Bucharest, 1–7 Polizu St., 011061 Bucharest, Romania; 2 Department of Public Health, Faculty of Dental Medicine, “Carol Davila” University of Medicine and Pharmacy, 8 Eroii Sanitari Blvd, 050474 Bucharest, Romania; 3 School of Life Sciences, Ecole Polytechnique Fédèrale de Lausanne (EPFL), Route Cantonale, 1015 Lausanne, Switzerland; titotu@student.ethz.ch (T.T.); rm.buga@yahoo.com (R.-M.B.); 4 Department of Dental Techniques, Faculty of Midwifery and Nursing (FMAM), “Carol Davila” University of Medicine and Pharmacy, 8 Eroii Sanitari Blvd, 050474 Bucharest, Romania; corina.cristache@umfcd.ro; 5 Department of Biotechnology, Faculty of Science, Necmettin Erbakan University, Yeni Meram Boulevard Kasim Halife Street, Meram, Konya 42090, Turkey; ferci@erbakan.edu.tr; 6 Biotechnologies Center, University of Agriculture and Veterinary Medicine, 42 Blvd. Mărăşti, 011464 Bucharest, Romania; camelia_crst@yahoo.com; 7 Department of Bioengineering, Yildiz Campus Barbaros Bulvari, Yildiz Technical University, Istanbul 34343, Turkey; iisildak@yahoo.com

**Keywords:** hyaluronic acid membrane, pH influence, melatonin, tetracycline, metronidazole, pH stability control, kinetic mechanism

## Abstract

(1) Background: The standard treatment for periodontal disease, a chronic inflammatory state caused by the interaction between biofilms generated by organized oral bacteria and the local host defense response, consists of calculus and biofilm removal through mechanical debridement, associated with antimicrobial therapy that could be delivered either systemically or locally. The present study aimed to determine the effectiveness of a hyaluronic acid membrane matrix as a carrier for the controlled release of the active compounds of a formulation proposed as a topical treatment for periodontal disease, and the influence of pH on the complex system’s stability. (2) Methods: The obtained hyaluronic acid (HA) hydrogel membrane with dispersed melatonin (MEL), metronidazole (MZ), and tetracycline (T) was completely characterized through FTIR, XRD, thermal analysis, UV-Vis and fluorescence spectroscopy, fluorescence microscopy, zeta potential and dielectric analysis. The MTT viability test was applied to check the cytotoxicity of the obtained membranes, while the microbiological assessment was performed against strains of *Staphylococcus* spp. and *Streptococcus* spp. The spectrophotometric investigations allowed to follow up the release profile from the HA matrix for MEL, MZ, and T present in the topical treatment considered. We studied the behavior of the active compounds against the pH of the generated environment, and the release profile of the bioactive formulation based on the specific comportment towards pH variation. The controlled delivery of the bioactive compounds using HA as a supportive matrix was modeled applying Korsmeyer–Peppas, Higuchi, first-order kinetic models, and a newly proposed pseudo-first-order kinetic model. (3) Results: It was observed that MZ and T were released at higher active concentrations than MEL when the pH was increased from 6.75, specific for patients with periodontitis, to a pH of 7.10, characterizing the healthy patients. Additionally, it was shown that for MZ, there is a burst delivery up to 2.40 × 10^−5^ mol/L followed by a release decrease, while for MEL and T a short release plateau was recorded up to a concentration of 1.80 × 10^−5^ mol/L for MEL and 0.90 × 10^−5^ mol/L for T, followed by a continuous release; (4) Conclusions: The results are encouraging for the usage of the HA membrane matrix as releasing vehicle for the active components of the proposed topical treatment at a physiological pH.

## 1. Introduction

Controlled drug release, vital for pharmaceutical therapies in healthcare, is a dynamic, either pulsatile or extended-release of active compounds to avoid side effects and maintain a constant, topical, or plasma level of the medication in chronic diseases [1,2,3,4,5,6].

Periodontal disease is a chronic inflammatory state caused by the interaction between biofilms generated by the organized oral bacteria and the local host defense response [7,8].

Following the complex physiological processes involved in the periodontal tissue damage, the bone is lost, minerals as calcium ions are released, and the periodontal pocket is formed [9].

The standard treatment for periodontitis consists of calculus and biofilm removal through mechanical debridement, associated with antimicrobial therapy that could be delivered either systemically or locally [10]. Additionally, specific inflammatory parameters such as bleeding on probing (BOP) and probing depth (PD) could be reduced through chemotherapeutical treatment, which improves the healing process by reducing bacterial load and inflammation [11,12].

Although saliva contains a significant amount of water (over 90%), it remains a complex electrolyte due to the contributions of submandibular, sublingual, and other salivary glands [8,13], as well as serum, leucocytes, microorganisms, etc. Consequently, there is a wide concentration range of inorganic and organic compounds that makes the resting (pooled) saliva a very complex oral environment. Usually, saliva is typically characterized by a pH range between 6.3 and 7.8, although studies prove that the resting pH range is much more extended, from 5 to 9 [14]. Much more, it has been shown that the pH values differ significantly between periodontal pockets and even between readings from the same pocket. Such observations demonstrated that there are very complex physiological processes involved [14,15]. In healthy subjects, saliva succeeds in maintaining a pH around 7.0 for healthy patients (pH = 7.10) due to its actions in controlling bacterial activities and neutralizing the acidity through its buffering power. When acidemia is present in the oral environment, pH lower than 7.0, then various oral diseases such as periodontitis could affect the patient. Local or topical treatment is preferred over systemic treatment due to significantly lower side effects and less patient compliance [10]. The commonly used topical antimicrobial agents are chlorhexidine, metronidazole, tetracycline, ciprofloxacin, doxycycline and minocycline [16,17,18,19]. One of the active antimicrobial agents, MZ, is already used for local periodontal treatment, alone or in combination with other antibiotics, such as tetracycline. Some studies have presented improved delivery methods of MZ in such a way to improve its local action [20]. MZ, C_6_H_9_N_3_O_3_, 2-Methyl-5-nitro imidazole-1-ethanol, is indicated in generalized periodontitis with positive detection for *Porphyromonas gingivalis*, *Bacteroides forsythus*, *Treponema denticola*, or *Peptostreptococcus micros* [21,22]. Another frequently used antimicrobial agent, tetracycline-C_22_H_24_N_2_O_8_-(4S,6S,12aS)-4-(dimethylamino)-1,4,4a,5,5a,6,11,12a-octahydro-3,6,10,12,12a-pentahydroxy-6-methyl-1,11-dioxo naphthacene-2-carboxamide, inhibits the activity of collagenase in the gingival tissue affected by periodontitis, thus exerting its modulatory effects on the host [23]. For instance, when using T fibers for local treatment of periodontal wounds, an important reduction in the probing depth of about 14% was recorded [24]. In the meantime, considering that systemic antibiotic delivery generates side effects, leading to increased bacterial antimicrobial resistance (AMR) [25,26,27,28], the local administration of the suitable drugs that allow direct and controlled delivery inside the periodontal pocket appears to be a wise and appropriate treatment option. A common adjunctive topical treatment for periodontitis contains a mixture of two antibiotics, T and MZ, in a white Vaseline vehicle, known as TM paste [29]. The last-mentioned component, white Vaseline (also known as petroleum jelly)-1,1,2-Trimethylbenzeindole, has proved to have healing properties when used in topical applications. Although the TM paste is used like a topical antimicrobial treatment, the outcomes are not satisfactory. Moreover, for increasing efficiency of the topical treatment applied, a constant level of active compounds need to be released into the periodontal pocket for a specific time period.

Additionally, tetracycline and metronidazole are mainly antimicrobial agents. Therefore, we have proposed a new type of topical treatment in which, besides antibiotics, some other active components for tissue repair, reducing local inflammation and improving the antibacterial activity were introduced. Such biologically active and biocompatible components could be melatonin (MEL) and hyaluronic acid (HA), based on their specific biological actions.

One of the chemotherapeutical anti-inflammatory agents, MEL, has been intensively studied lately, based on its lack of toxicity, structural, and physiological specificities [30,31,32,33,34,35]. Therefore, in the case of periodontal compromised patients, clinical studies were performed using MEL as supportive therapy after scaling and root planning (SRP) [36,37,38,39]. However, the effect of MEL in a complex active topical mixture was not sufficiently investigated [40]. MEL, C_13_H_16_N_2_O_2_, *N*-acetyl-5-methoxy-tryptamine, is an indole amine, a natural hormone that is produced in a circadian manner by the pineal gland and in smaller quantities by the brain, bone marrow, retina, ciliary body, thymus, placenta, gonads, gastrointestinal tract, and skin [41,42,43]. Most of the MEL involved in physiological and pathological processes is generated by the pineal cells, where the sequential action of arylalkylamine *N*-acetyltransferase and hydroxy indole-*O*-methyltransferase on serotonin takes place on tryptophan, its precursor [44,45]. The MEL biochemistry evidenced its primary degradation through the indol pathways [46,47]. Among its specific physiological functions, MEL presents anti-inflammatory action and antioxidant behavior [48,49,50,51,52]. Thus, Reiter et al. [53] showed that MEL acts as a scavenger for reactive oxygen and nitrogen species, limiting oxidative stress by stimulating the antioxidant enzyme. It was shown that MEL is essential in the processes involved in bone formation and the limitation of bone resorption [54,55]. Carpentieri et al. [46] evidenced the passive diffusion of MEL from the systemic circulation into resting saliva when used in periodontal therapy, recording a range of MEL concentrations in plasma from 24% to 33%.

Casale et al. [56] identified the presence of hyaluronic acid in various quantities in both non-mineralized (periodontal ligament, gingiva) and mineralized tissues (alveolar bone). HA presents a beneficial action in wound healing, tissue injury repair, immunosuppression [57,58,59,60,61]. The linear poly-anion, structurally formed by a repeating disaccharide structure [(1→3)-β-d-GlcNAc-(1→4)-β-d-GlcA-] [62] that is the HA, was first isolated from the vitreous humor of cow’s eyes. The HA is also naturally present in the extracellular matrix of connective tissue, skin, vitreous humor, synovial fluid, and many other tissues and organs of the body, accounting for 15 g in the case of a 70 kg adult [63]. Starting from the studies of Pagnacco et al. [64] who assessed the antibacterial, anti-edematous, and anti-inflammatory effects of HA when used as a topical treatment for periodontitis in a clinical trial, we decided to use HA as a matrix for antibiotics and MEL. Additionally, in the gingival crevicular fluid, a naturally high level of HA was determined, while it was absent in cases of gingivitis or periodontitis [41]. Thus, it is possible to evaluate the presence and degree of periodontitis considering HA as a marker. Some studies highlighted reduction in the periodontal tissue inflammatory process when HA was used [65,66,67].

A bioactive compound such as HA that has a proven action in reducing inflammation, and then in the osteoblast formation processes, may increase the efficaciousness of the antibacterial behavior of topically applied antibiotics. Consequently, it is expected that a topical formulation consisting of MEL, MZ, and T will be more effective in treating periodontitis when dispersed into a HA matrix. In an extensive and well-sustained review, Bayer [68] presented the applications of HA as a carrier for bioactive compounds and a delivering matrix for various drugs’ controlled release. In the actual context of the high interest in controlling the antimicrobial resistance (AMR) global phenomenon, it is necessary to find viable solutions for assuring the antibiotics’ minimum inhibitory levels and their controlled release over an adequate treatment period. The specific physical and chemical characteristics of HA recommend it as matrix for controlled release of antibiotics, antimicrobials, or antiseptics [69,70,71,72,73,74]. In such a context, our proposed method for loading biocompatible material, such as HA, with antibiotics, could offer the possibility to treat efficiently limited targeted areas, avoiding the side effects.

We proposed using the unmodified HA for controlled delivery of active topical treatment with action on periodontal pathogens, with anti-inflammatory and regenerative capacity. The new topical treatment contains antibiotics, T and MZ, and an anti-inflammatory chemotherapeutical agent such as MEL [75]. Our previous research, which allowed for the development of a patent application, proved the stability and synergic action of the active compounds within the proposed topical treatment [76,77]. The achievement of the combination of the active compounds was followed by the evaluation of cytotoxicity and antibacterial activity. In this study, the HA and MEL, MZ, T were associated for several reasons: mixing HA and MEL can accelerate the periodontal healing process; the proposed complex material protects the wounds from contamination and infection due to the presence of antibiotics; HA also is expected to present a synergistic effect on the antibacterial activity of the mixture. The introduced releasing membrane in this investigation is a new approach, aiming to accelerate the process of wound healing in completion of antibacterial treatment. The ultimate purpose of using HA as a releasing membrane for the active compounds is its potentially usage as a topical wound treatment in periodontitis, improving and accelerating the overall tissue-healing process. In the meantime, the present study aimed to evaluate the releasing profile against saliva pH for the mixture containing the three active compounds, T, MZ, and MEL, using the HA as matrix.

## 2. Materials and Methods

### 2.1. Preparation of the Bioactive Formulation

The freshly prepared solutions of MEL, C_13_H_16_N_2_O_2_, N-acetyl-5-methoxy-tryptamine (MEL—Sigma-Aldrich, St. Louis, MO, USA), were obtained in ethanol (Merck KGaA, Darmstadt, Germany). The high-purity chemicals were provided by Sigma-Aldrich, Merck, Germany: HA (HA) of 300kDa; C_6_H_9_N_3_O_3_-1-(2-Hydroxyethyl)-2-methyl-5-nitro-imidazole, MZ (MZ); and C_22_H_24_N_2_O_8_-(4S,4aS,5aS,6S,12aS)-4-(Dimethylamino)-3,6,10,12,12a-penta hydroxy-6-methyl-1,11-dioxo-1,4,4a,5,5a,6,11,12a-octahydro tetracen-2-carboxamid-T (T). All chemicals were analytical grade: methanol and ethanol, hydrochloric acid 0.1 M, sodium hydroxide 0.1 M, sodium phosphate dibasic dihydrate, sodium phosphate monobasic monohydrate, sodium bicarbonate, citrate and glycine, potassium chloride (Merck KGaA, Darmstadt, Germany) were also used. All necessary chemicals to prepare the artificial saliva were procured as analytical-grade reagents from Sigma-Aldrich, Merck, Germany (Merck KGaA, Darmstadt, Germany): KCl, MgCl_2_·6H_2_O, CaCl_2_·2H_2_O, K_2_HPO_4_, KH_2_PO_4_, methyl-p-hydroxybenzoate, and sodium carboxymethyl cellulose. All solutions were prepared in deionized water (conductivity 1.28 µS/cm^2^) obtained on a Milli-Q system (Sartorius GMBH, Gottingen, Germany). For homogenization of the active combinations, an ultrasonic bath (Elmasonic S10 H, Elma Schmidbauer GmbH, Singen, Germany) was used for solutions and a trituration mill (Retsch-PM 100) for solids. The necessary pH measurements were performed using a Hanna Laboratory Multiparameter HI5522-02 with pH electrodes (HI10530, HI1343B) (Hanna Instruments Romania, Cluj-Napoca, Romania).

**Figure 1:**
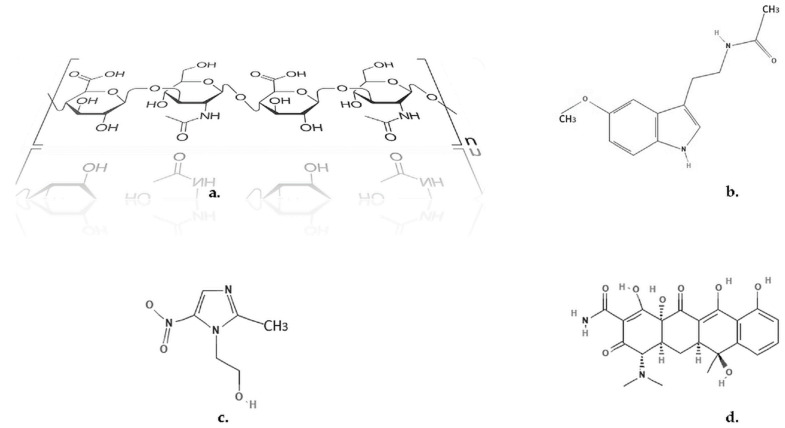
Chemical structures. (**a**) HA; (**b**) MEL; (**c**) MZ; (**d**) T [79,80,81].

The complex mixture containing T, MZ, HA (each 3% each), and MEL (1.8%) (Figure 1) was prepared in two different containers. Water (89.2%) was added into one of the containers and mixed vigorously manually and into an ultrasonic bath. By adding the water to the mixture containing HA it was obtained the gel. This hydrogel phase also included the dissolved active compounds. The solid amount in the other container was mixed thoroughly (solid trituration), but no water was added. The mixtures were then poured into brown tubes, sealed, and stored in dark and cool (−20 °C) conditions [78]. The membrane-like hydrogel was made by spreading a thin layer into a Teflon support used to prepare the ion-selective membranes, and pressing the formed film with an appropriate Teflon lid. Finally, membranes of the complex bioactive hydrogel matrix were obtained with a width of 0.5 mm. These membranes were used to follow up the releasing profile of MEL and antibiotics.

### 2.2. Physicochemical Characterization of the Bioactive Formulation

The thermal analysis was performed on DuPont 910 DSC equipment (DuPont, DuPont Instruments, Wilmington, DE, USA) at a heating rate of 20 °C/min, over a wide temperature range (room temperature up to 650 °C) using ceramic pans. Thermal analysis of the mixtures was performed under a nitrogen atmosphere, using the samples in two different forms: gel (hyaluronic gel) and powder (solid).

Structural Analysis was performed through FTIR spectrophotometry and XRD analysis. The FTIR investigation was run on a Brucker Tensor 27 (ATR) system (Brucker Optics, Ettlingen, Germany) at a spectral resolution of 4 cm^−1^, covering a 500–4000 cm^−1^ range; the OPUS NT 7.0 (Brucker Optics, Ettlingen, Germany) software was applied. XRD measurements were performed using PANalytical X’Pert Pro X-Ray diffractometer (Malvern Panalytical Ltd., Malvern, UK) equipped with Ni-filtered CuKα_1_ (λ = 1.54060 Å) operated at 40 mA, 45 kV in the range of 2θ from 5° to 100° with a step size of 0.03°/s.

The UV-Visible spectra for bioactive forms were recorded using a dual-beam spectrophotometer T80+, PG Instruments (PG Instruments Limited, Leicestershire, UK) using 10 mm quartz cuvette. The scan range was between 200 and 700 nm, the interval was 1.0 nm, and a medium speed was selected. The UV-Vis spectrophotometric analysis for each active compound was performed and presented in our previous works [75,77]. The fluorescence experiments were run on a PerkinElmer LS45 Luminescence Spectrometer (PerkinElmer Inc., Waltham, MA, USA) in 10 mm × 10 mm quartz cuvettes with excitation wavelength at 278 nm (for MEL) and 340 nm (for T) at bandwidth (Ex): 5 nm/bandwidth (Em): 20 nm, response time set to 0.02 s at a medium sensitivity. The scanning speed was 200 nm/min.

The preservation of the autofluorescence characteristics of some of the bioactive components, MEL, and T, was investigated through fluorescence microscopy. The experimental part has been performed on Leica DM 3000 LED microscope (Leica Microsystems, MEDIST Life Science, Bucharest, Romania). The image acquisition was conducted using a reduced-noise-factor camera (MC190 HD, Leica Microsystems) and a fluorescence metal–halide lamp (EL6000, Leica Microsystems) taking the advantage of the toggle mode, automated correct positioning and light-intensity adjusting features.

For checking the active mixture stability, electrochemical measurements were performed. The determination of the Zeta potential of the mixture was measured in an aqueous solution at a pH = 7.0, at room temperature 25 °C, using a Zetasizer Nano-ZS (Malvern Panalytical Ltd., Malvern, UK). The conductivity was 0.0385 mS/cm. Additionally, electrochemical parameters such as ionic conductivity and ionic viscosity were investigated with the help of the dielectric analyzer—DEA 288—Netzsch (NETZSCH-Gerätebau GmbH, Selb, Germany) using a detection-sensor-type Coated Tool Mountable Comb Electrode (NETZSCH-Gerätebau GmbH, Selb, Germany). The measurements were performed at 1 kHz. The bioactive material thicknesses were 0.5 mm.

### 2.3. Cytotoxicity Analysis

The cytotoxicity analysis of the active mixture, both in gel and solid form, was performed applying the MTT viability test [82]. The MTT assay and Dulbecco’s Modified Eagle Medium (DMEM) were procured from Thermo Fisher Scientific.

The photometric readings were taken at 570 nm. The hydrogel sample was prepared by dissolving it in DMEM to obtain a final concentration of 0.6 g/mL. The powder sample was also prepared in DMEM when a 0.01 g/mL final concentration was obtained. Mouse fibroblast cell line L929 (Sigma-Aldrich Merck KGaA, Darmstadt, Germany) was cultured in a 96-well plate (10^5^ cell/mL). Samples were held in wells for 24 h for interaction with the cells. The cells were left at 37 °C in a CO_2_ incubator for 24 h, and afterward, the samples were added to the wells. After one day, the MTT viability test was carried out. Furthermore, 1% phenol solution (Aldrich, Merck KGaA, Darmstadt, Germany) was used as a positive control, while the culture environment was used as a negative control.

The experimental results obtained in triplicate (n = 3) are presented as mean ± standard error of means. One-way ANOVA method was applied (IBM^®^ SPSS^®^ 18.0 statistical software, Armonk, NY, USA), followed by Tukey’ post hoc test. Significance was set at a *p*-value < 0.05.

### 2.4. Microbiological Assessment

#### 2.4.1. Reactivation of Microorganisms for the Minimum Inhibitory Concentration (MIC) Testing

Bacterial strains from stock culture were reactivated by inoculation on the blood agar medium. After overnight incubation at 37 °C, isolated colonies were selected, and organism identity was confirmed. Isolated colonies were transferred to sterile brain–heart infusion (BHI) (Merck KGaA, Darmstadt, Germany) liquid medium for bacteria and incubated once more overnight. Using McFarland’s 0.5 turbidities standard scale, the growth concentration was adjusted to 10^5^ organisms/mL.

#### 2.4.2. The Minimum Inhibitory Concentration (MIC) Antibiogram

A volume of 180 µL of brain cord infusion broth (BHI) was added to each of the five tubes/concentration/MIC bacterial strain. In the first MIC tube containing 200 µL broth, 20 µL of AS (active substance) was added. Different batches of AS were studied, namely MEL, HA, MZ, T, their solid mixture, and their combination with soft paraffin (Sigma-Aldrich, Merck, KGaA, Darmstadt, Germany) which could be considered as an alternative release vehicle for the bioactive compounds, and allows a comparison with the TM paste.

After a functional homogenization, 200 µL was transferred to the second MIC tube, and the procedure was applied to all five tubes. From the last tube, the 200 µL final solution was removed. Following this dilution in series, the AS concentrations obtained were 2500, 1250, 625, 312.5, and 156.25 µg/mL. For each of the five MIC tubes with different concentrations, 20 µL of the previously prepared strains of *Staphylococcus* spp. and *Streptococcus* spp. were added. The final volume/tube was 200 µL. After incubation, MIC values were determined by visual examination of the tubes and assessing dehydrogenase activity. For each batch of tests, positive and negative controls were placed in the brain–heart culture infusion culture medium. The bacterial strain showed turbidity for positive control, while the negative control containing only the brain–heart infusion culture medium remained clear. In each series of tubes, the first tube with clear supernatant was considered without any growth and was taken as a specific MIC value for the sensitive strain. When turbidity is present in a MIC tube, it indicates an increase in bacteria, signifying that the organisms were resistant to the active substances tested.

### 2.5. pH Influence on the Stability of the Active Compounds

In order to establish the optimal pH range for the study of the controlled release of the bioactive mixture through the HA hydrogel membranes, experimental studies of the influence of pH on the activity of each bioactive component were performed.

A standard stock solution of each bioactive component, MEL, MZ, and T, was prepared in an adequate solvent. Before each analysis, the standard stock solutions were freshly obtained. The pH influence on the stability of each active compound was assessed for one week. The rationale behind the 7-day time frame is based on the duration of the topical treatment proposed, which should be applied once a week and repeated for 60 days according to the protocol for the clinical trial ClinicalTrials.gov Identifier: NCT03656484. The pH values were measured for each sample after certain storage time to assure no significant pH change occurred. All experiments were triplicated.

For MEL, the 100 μg/mL stock solution was prepared in methanol, and it was subsequently diluted to obtain five different concentrations (10^−3^ μg/mL–10 μg/mL) used for the calibration curve. The standard stock solution of MEL was added as 1 mL into phosphate-buffered solutions that were adjusted at pH 2, 4, 6, 7, and 9 using 0.1 M HCl and 0.1 M NaOH. Then, 10 mL of MEL solution was introduced to 10 mL brown graduated flasks and stored in dark conditions at room temperature. To assess the MEL concentration every day for one week, the solutions were taken out and measured spectrophotometrically at the same moment of the day. An aliquot of each sample was taken up in a quartz cell (1 cm) and introduced to the fluorescence spectrophotometer (Perkin-Elmer LS45 Luminescence Spectrometer) to determine the remaining MEL concentration. The readings were performed for the maximum from 570 nm when the excitation wavelength was 278 nm.

The T molecule possesses four potentially dissociable protons: keto-phenolic hydroxyl groups, tricarbonyl-methane, and dimethyl-amino groups. The multiple functional groups grafted on T transformed the antibiotic into an amphoteric compound [83]. Thus, pH becomes an essential factor affecting the stability of T in the solution. The concentration of T was determined every day for one week, at the exact same moment of the day, through fluorescence spectrophotometric analysis at 335 nm excitation wavelength, and reading the maximum at 530 nm. The buffer solutions, 10^−2^ mol/L, used during the determinations were prepared as follows: for pH = 2, glycine with HCl; for pH = 4 and pH = 6, citrate buffer; for pH = 7 and pH = 9, phosphate buffer. The standard stock solution of T, 50 μg/mL, was prepared using ethanol. Then, the T standard solution was subsequently diluted with buffers according to the described procedure for preparing and storing the MEL samples. The 50 μg/mL T stock solution was used to obtain the five different concentrations (10^−3^ μg/mL–10 μg/mL) needed for the calibration curve.

The third bioactive component used, MZ, undergoes hydrolysis reaction, in presence of hydroxyl radicals [84]. Therefore, it is important to follow the behavior of MZ solution at various pH values. A stock solution of 50 μg/mL MZ was prepared in deionized water. For the calibration curve, the stock MZ solution was diluted to prepare five different concentrations between 10^−3^ μg/mL–10 μg/mL. For the study of the pH influence on the possible MZ degradation, particular volumes of the stock MZ solution were diluted into an appropriate amount of adjusted buffer solution. The sample solutions were stored in 10 mL brown graduated flasks and put in dark conditions at room temperature. The buffer solutions (10^−2^ mol/L) used were prepared as shown above for T. The concentration of MZ was assessed each time the other bioactive compounds were determined, i.e., every day for one week, through UV spectrophotometric analysis following the variation in absorbance at 320 nm.

The ionic strength influences the activity coefficient, thereby causing errors in measurements. Therefore, the ionic strength of the sample solutions must be kept constant. In order to achieve this, an indifferent salt (supporting electrolyte like KCl)—which does not react with the target bioactive compounds—needs to be added to the sample. Consequently, the constant ionic strength was maintained at 0.5 by adjusting the quantity of potassium chloride in solution.

### 2.6. Release Profile Measurements

**Figure 2:**
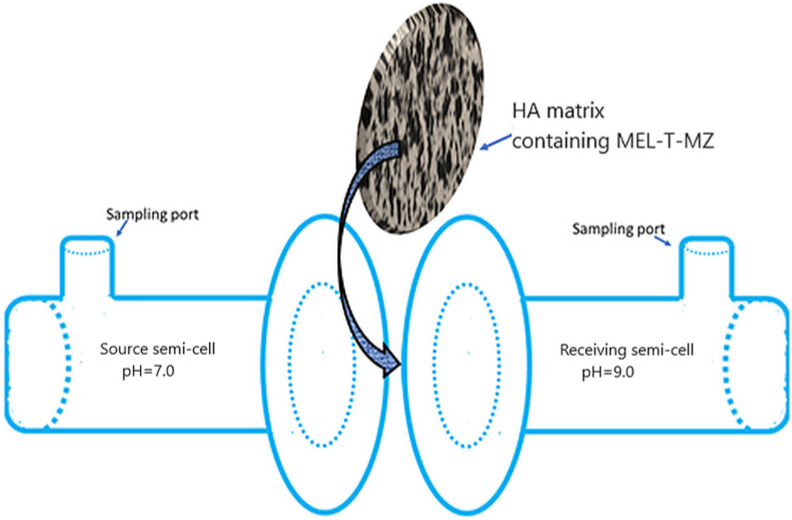
Homemade horizontal-flow diffusion cell.

The experiments for releasing the bioactive components of the complex mixture, MEL, T, and MZ, through the biocompatible membrane formed by the HA matrix were performed using a homemade glass horizontal-flow diffusion cell (Figure 2). The HA membrane was prepared as mentioned above in Section 2.1. The diffusion cell was composed of two chambers, each with 20 mL capacity, that were closely clamped. The solutions were introduced in the two chambers through separate sampling ports to avoid any concentration polarization. Subsequently, they were stirred with the aid of a magnetic stirrer (HI190M, Hanna Instruments Romania, Cluj Napoca, Romania) and tiny magnetic bars introduced into the chambers. As shown in Figure 2, the hydrogel matrix membrane-like structure introduced between both chambers/semi-cells secured the cell. In order to differentiate the semi-cells between them, depending on the feeding electrolyte, they were assigned as source and receiving chambers/semi-cells. The buffered saliva at pH = 7.0 was introduced into the source chamber, while the receiving chamber was filled with saliva at pH = 9.0. The rationale behind choosing different pH values for the two parts of the diffusion cell is based on the reported experimental observations that the pH of the gingival crevicular fluid (GCF) is highly basic [85], far from the neutral range of saliva. Consequently, it is of interest to determine the amount of released active formulations when the immobilizing membrane (HA hydrogel) is facing electrolytes with different pH values. The used artificial saliva was prepared using KCl (8 × 10^−2^ moles), MgCl_2_·6H_2_O (2.9 × 10^−4^ moles), CaCl_2_·2H_2_O (5.6 × 10^−4^ moles), K_2_HPO_4_ (4.6 × 10^−3^ moles), KH_2_PO_4_ (2.4 × 10^−3^ moles), methyl-p-hydroxybenzoate (1.3 × 10^−2^ moles), and sodium carboxymethyl cellulose (3.8 × 10^−2^ moles) in 1 L deionized water. The pH during the determinations was maintained at specific values in the two chambers of the diffusion cell using adjusted Tris-buffer (Millipore, Merck KGaA, Darmstadt, Germany). During the experiments, no leaking of chamber fluids took place. The concentration changes of the active components (MEL, T, MZ) were monitored spectrophotometrically. At different times, 1 mL samples were taken from each chamber, and the concentrations of the bioactive components were measured. For each determination, another diffusion cell with the same dimensions was used. The migration of the active components through the HA hydrogel membrane was studied through the UV-Vis spectrophotometric technique.

The UV-Vis spectrophotometric investigations were run on a dual-beam spectrophotometer Varian Cary^®^ 50 (Victoria, Australia) UV-Vis. The spectral bandwidth was 1.5 nm at a resolution of 1 nm and a scan rate of 300 nm/s, over wavelength range λ ∈ (200 to 800 nm). Quartz cells of 10 mm were used. The working temperature was 25 °C.

As it is known, the active formulations are released mainly through diffusion from the hydrogel matrix. There are a few release kinetics models that could allow us to find the best fit for the experimental data. Depending on the associated model, it is possible to issue rationales on the release mechanisms. In general, the release mechanisms governed by various kinetics of bioactive components are not accurately described by models. Such failure could be assigned to the fact that the releasing surfaces were considered plane, while the specific curvature imparts different characteristics than expected. If the HA hydrogel was considered as a flat system, then the drug diffusion could be modeled through a one-dimensional mechanism, applying the second law of Fick [86,87]. However, in the case of the obtained HA hydrogel, there is a three-dimensional structure [88]. Thus, the Korsmeyer–Peppas predictive model [89] is most likely to provide an appropriate design of the drugs’ delivery.

The most common kinetic model applied was developed by Korsmeyer and Peppas, and is based on the relationship [89]:

(1) $$\frac{M_{t}}{M_{\infty }}=\;k\cdot t^{n}$$

where M_t_ is the total quantity of a drug released at a time t, M_∞_ is the total quantity of dispersed drug into the matrix that should be released, n is the diffusivity exponent, and k stands for the kinetics constant.

If we consider that a Fickian diffusion process takes place in the HA matrix, then:

(2) $$k=\sqrt{\frac{4D}{\pi \cdot L^{2}}}$$

where D is the diffusion coefficient of a certain drug, L is the half thickness of the membrane (HA hydrogel). For a Fick-type diffusion, the diffusivity coefficient is equal to 0.45, while for a non-Fick-type diffusion it has a value ranging between 0.45 and 0.89 with an anomalous behavior when n has unitary value. When n = 1, there are zero-order kinetics in place, and the swelling of the polymer is considered the trigger factor of drug release. In general, Fick-type diffusion occurs through a rubbery polymer such as the hyaluronic hydrogel, depending on the water behavior in the polymeric matrix and the specificity of the diffusion rate [90].

The Higuchi model [91], which was also used, introduces a simplified equation compared with (2), as it considers a value of 0.5 for the diffusivity constant, n. Thus, the equation is:

(3) $$\frac{M_{t}}{M_{\infty }}=\;k\cdot \sqrt{t}$$

The above model was successfully applied to model planar delivery systems and in the case of the drug releasing under a predominantly diffusion-based process, developed inside the three-dimensional matrices [92].

Usually, zero-order kinetics (4) and first-order kinetics (5) models are also used, for which the following equations apply:

(4) $$\frac{M_{t}}{M_{\infty }}=\;\mathrm{kt}$$

(5) $$\frac{M_{t}}{M_{\infty }}={\;e}^{-k\cdot t}$$

The kinetic studies provide a better understanding of the release process, namely the drugs’ behavior in the presence of distinct bioactive components [93]. Consequently, the release of MEL, MZ, and T from the HA hydrogel membrane was studied using the above equations.

The correlation coefficient (R^2^) was used to establish the best-fitting kinetic model to the experimental data:

(6) $$R^{2}=\frac{\sum {\left(M_{m}-\bar{M_{e}}\right)}^{2}}{\sum {\left(M_{m}-\bar{M_{e}}\right)}^{2}+\sum {\left(M_{m}-M_{e}\right)}^{2}}$$

where M_e_ is the amount of the released drug at equilibrium obtained from experimental data, M_m_ is the releasing capacity—a constant obtained from the kinetic model—and $\bar{M_{e}\;}$ is the average of M_e_. 

## 3. Results

### 3.1. Physicochemical Characterization of the Bioactive Formulation

The results of the thermal analysis results of the active compounds in the HA gel matrix are shown in Figure S1. The hydrogel mixture owes 89.2% of its weight to water, clearly shown on the TG curve—Figure S1a. However, after the vaporization of the water, there seems to be a very slight change in the mass of the active mixture, seemingly not resembling the thermal behavior of the powder form—Figure S1b. This is due to the high water content that was evaporated in the first stage. DTA showed almost no changes other than a substantial endothermic peak, indicating that evaporating water is also related to high water content. Additionally, the large endothermic peak at 100 °C may be attributed to the melting of the active components. The DTG curve shows tiny changes before 200 °C and around 250 °C, which is somewhat similar to the thermal behavior of the solid form of the mixture and might indicate some overlapping decomposition process that could be possibly visualized if a much lower heating rate (1.25 °C/min) is applied. Thermal evolution up to 200 °C highlighted a mass loss of the gel form (around 9%) that was almost identical to that of the powder mixture (about 7%) over the same temperature range. The mass loss gradually continues after 300 °C, as can be seen on the thermal diagram. At the end of the thermal analysis, the amount of residue is 4.7%. In comparison, the solid mixture had a residue of 37.4%—Figure S1b. An interaction between HA and the other solid materials may play a role in this large difference between the residue amounts. It is very likely that the insoluble matter, as solid impurities present in MZ, T and MEL, play a role in the much higher remaining residue when the solid mixture was thermally analyzed. The impurities in HA are of protein origin, therefore are unlikely to contribute to the final sold residue. A possible explanation for the significant increase in the thermal stability of the powder mixture could be attributed to the HA chains that might encompass the active compounds. Consequently, a higher energy would be necessary to release the MEL, MZ and T moieties. In Figure S1b, there are two endothermic peaks; the first at 86 °C is due to MEL melting point. Additionally, a loss of a small amount of adsorbed moisture corresponding to a mass loss of 5.7% is recorded. The second endothermic peak at around 165 °C could assigned to the melting points of MZ (160 °C) and T (170 °C). The range 160 °C–210 °C is characterized by more than a single thermal event according to the DSC curve, and a mass loss of 7.4%, which may imply the beginning of the first stage of decomposition for HA. According to the literature, this decomposition of HA continues toward 300 °C, and the second stage of its decomposition begins around that temperature [94]. The third endothermic peak at 230 °C on the DTA curve may be attributed to the HA melting point (241 °C), which is in good agreement with the literature data [94]. Deterioration of T starts after 210 °C, while MZ is decomposed in two exothermic steps at 290 °C and 375 °C, the latter probably being one of the reasons for a slight exothermal change in the DTA curve around that temperature. As mentioned above, the final residue amount was about 37.4%.

Figure S2 from the Supplementary Material File presents the FTIR spectra of the complex mixture. The first spectrum, Figure S2a, clearly shows the specific bands for OH (3267 cm^−1^), N-H (3094 cm^−1^), various C-H types (between 2800–3000 cm^−1^ (C-H stretching), around 1400 cm^−1^ (CH_3_ bending), around 1360 cm^−1^ (CH_3_ bending), 859 cm^−1^ (C-H out of plane deformation), and 739 cm^−1^).> Additionally, there are bands for C=C, C=O bonds, and C-O related amide (between 1600–1650 cm^−1^), C=C (1580 cm^−1^), C=N (between 1500–1550 cm^−1^), N=O (around 1480–1490 cm^−1^ and 1360 cm^−1^), O-H in COOH (1447 cm^−1^), amide (1325 cm^−1^), C-O-C (probably at 1222 cm^−1^), C-N (1067 cm^−1^), C-O stretching due to CH_2_-OH (at 1039 cm^−1^). At 946 cm^−1^ the bands specific to the ring asymmetrical out-of-plane bonds deformation are visible. Some of these bonds are present in more than one component of the mixture. As a consequence, various bonds in different components overlap around one band so their intensity may be higher than they would have been if the specific component was measured alone. It is highlighted that through the FTIR investigation it was possible to identify the specific absorption bands for each active compound, as they kept their identity within the mixed form [78].

The second FTIR spectrum of the complex hydrogel formulation, Figure S2b, evidenced a huge water effect on all bonds. All peaks that should be present in the functional group region between 1500 and 4000 cm^−1^ are totally drowned due to the presence of water. The water absorption bands in that region can be specifically observed. This could be explained by the formation of HA gel form following the incorporation of an important amount of water molecules. Peaks seem to be shifted in the fingerprint region, and peak intensities are also lowered because of both concentrations being low, and there is strong IR absorption of water throughout the measured spectrum. So, the latter FTIR reading is not meaningful for determining any difference between powder and hydrogel forms of the complex mixture.

**Figure 3:**
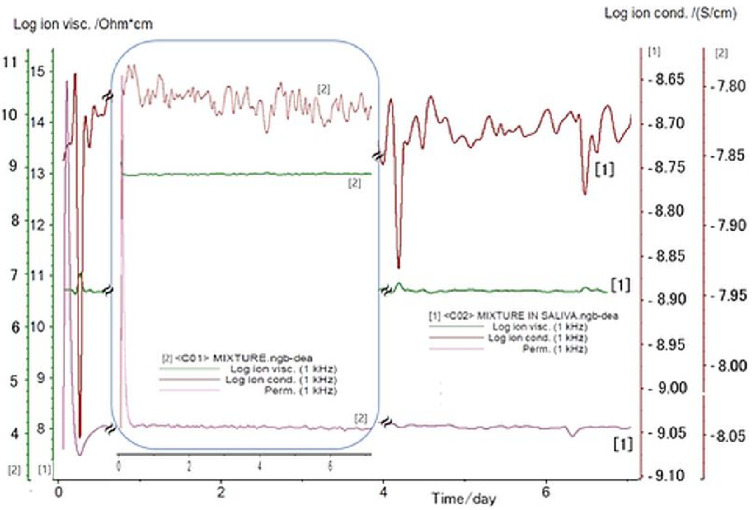
DEA analysis determined for the bioactive mixture immersed in saliva (1), and dry conditions (2).

The electrochemical Investigations are presented in Figure 3. The recorded data showed that the ionic viscosity increases when the bioactive mixture is immersed in saliva. Meanwhile, the ionic conductivity decreases from 1.77 × 10^−3^ (μS/cm) for dry composite to 1.41 × 10^−2^ (μS/cm) in the presence of saliva. These data are correlated with the ionic viscosity in saliva that increases by 32% compared to the dry conditions. Viscosity is an important feature associated with the material characteristic that affects the charge transport capacity. Any change in the viscosity of the bioactive composite could modify its specific transport properties, including conductivity, diffusion coefficient, or the charge transfer rate. The observed behavior could be assigned to the formed ions’ mobility. When incorporated into the HA gel, the specific structure of the bioactive compounds has an impact on the formation of hydrogen bonds and implicitly on the viscosity and ionic conductivity as well [95].

The presence of N-H and O-H functional groups on the structure of the considered biocomponents (Figure 1), MEL, MZ, T, and HA, imply both inter- and intra-molecular interactions through hydrogen bonding upon the formation of the hydrogel form through the dissolution in saliva. Consequently, the viscosity increases, while the ionic conductivity decreases. The experimental results comply with such behavior.

The UV spectrum given in Figure S3 shows the specific behavior of the complex bioactive material. Given that our sample material is a mixture, it is expected that we cannot see some bands due to their overlapping. However, after careful comparison with literature for each component individually [77], and the derivative spectrophotometric method [75], we identified the specific bands for the active compounds. MEL has an adsorption band at 278 nm wavelength and a shoulder at 301 nm [77]. HA sodium salt shows gradually decreasing absorptivity from 200 nm to 230 nm [76]. MZ has two adsorption bands at 232 and 321 nm [96], while the other antibiotic, T, has two broad bands at 275 and 360 nm wavelengths [97]. However, because of different conformations of T, the numbers of the maximum absorbance bands and wavelengths of the maximum absorptivity can be different [98]. The recorded spectrum presents the specific absorption bands that are compatible with the presence of each biocompatible compound.

**Figure 4:**
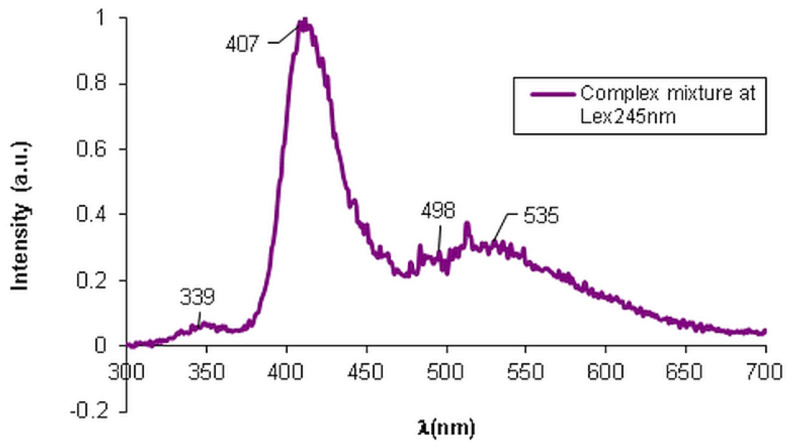
Fluorescence spectra of the complex mixture—HA, MEL, MZ, and T.

The fluorescence spectra presented in Supplementary Material, Figure S4a–d, highlights the maintenance of the fluorescence characteristic for each formulation when present in the mixture due to the synergic effect. The fluorescence intensity for both individual and mixture analyses was almost the same upon the usage of the excitation wavelength specific for each compound. However, in the case of MZ in mixture we noticed a shift in the emission band and a broader aspect of the maximum fluorescence intensity peak. Additionally, the presented spectrum shown in Figure 4 confirms the presence of MEL in a lower quantity compared to the other components, according to the fluorescence from 339 nm.

**Figure 5:**
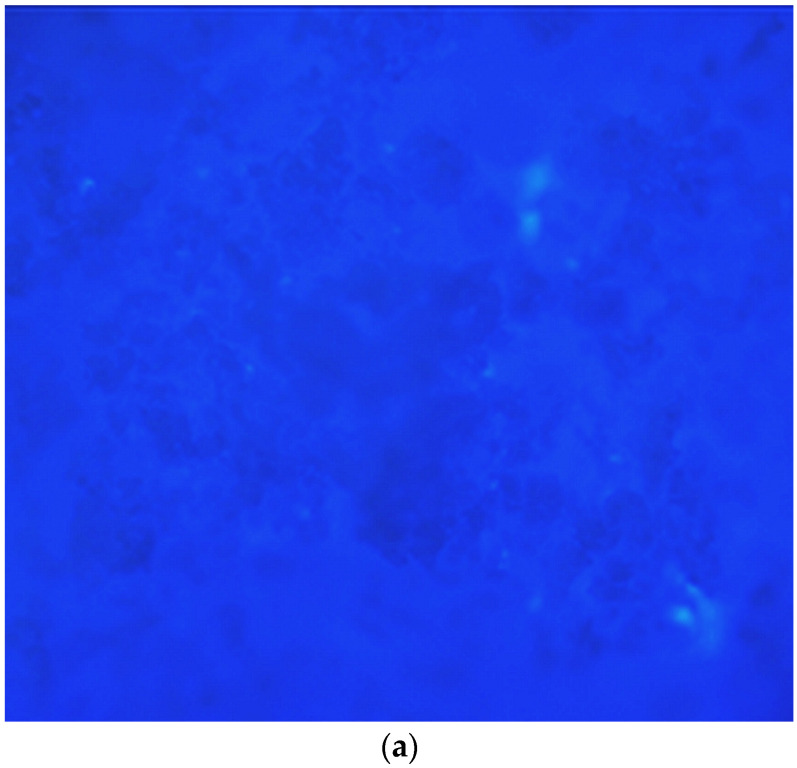

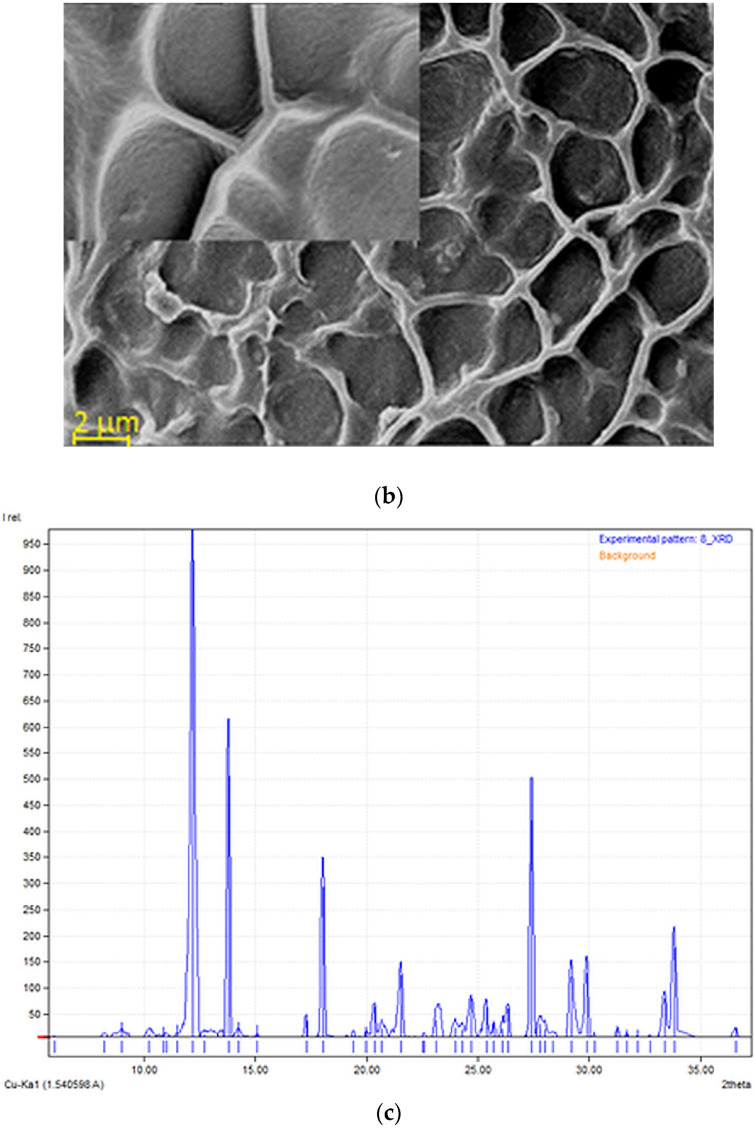
(**a**) Fluorescence microscopy image of the complex mixture of active formulations in HA hydrogel. (**b**) SEM image with included detail of the specific structure for the complex mixture; (**c**) X-ray diffraction pattern of HA-MEL-MZ-T mixture.

In Figure 4 presents the fluorescence spectra for the complex matrix containing MEL, MZ, T, and HA, when specific λ_ex_ = 245 nm was applied. Due to the exponential dependence of the fluorescence intensity, F, on concentration:

(7) $$F=k_{f}\cdot I_{0}\cdot \left(1-10^{-\varepsilon \mathrm{dc}}\right)$$

where k_f_ is the constant rate of the fluorescence process, I_0_ is the intensity of the incident radiation, ε is the absorption coefficient depending on λ_ex_ applied (L/mol·cm), d represents the optical path (cm) and c is the concentration (mol/L). Consequently, the fluorescence intensity of the components varies abruptly with the variation in their concentrations in the mixture. The obtained fluorescence spectrum of the bioactive matrix at λ_ex_ = 245 nm, that is specific for HA, highlights the presence of all components, with stress on the presence of HA with the same fluorescence intensity as in the individual HA fluorescence spectrum, but slightly shifted to 407 nm compared to the position of the maximum peak at 368 nm for the individual HA. The presence of T with an absorption band at 535 nm was observed. The absorption band from 498 nm was assigned to MZ, the characteristic wavelength being shifted from 502 nm for the singular MZ. A fluorescence process occurs when molecular excitation takes place with the impact with a photon within 10^−15^ s, followed by the excited electrons’ relaxation and the emission of the fluorescence radiation when the molecule is restored to its fundamental state. Such photochemical action upon a molecular system is used in the fluorescence microscopy that is largely used in clinical and biological determinations. This sensitive technique was used to investigate if the bioactive components from the proposed topical treatment preserve the physical–chemical individuality of the used formulations. The images of fluorescence microscopy presented in Figure 5a evidence the presence of the MEL autofluorescence that imparts a blue color to the HA hydrogel when the studied sample is irradiated with λ_ex_ = 278 nm. When the sample is irradiated with λ_ex_ = 450 nm, the other component of the complex mixture, T, with intrinsic fluorescence is excited, and it imparts a greenish color to the sample. As it is possible to identify the presence of these components through fluorescence microscopy, a pertinent conclusion could be drawn, namely that each component preserves its particular properties, such as the intrinsic fluorescence, although they are dispersed into a HA hydrogel matrix. Such an observation entitles us to consider that there are no structural changes that modify the characteristics of the components. Additionally, the fluorescence microscopy highlighted the open pores, the lace-like morphological structure of the HA hydrogel matrix that accommodates MEL, MZ, and T as active components.

It is known that the release profiles in terms of the specific kinetics depends on the diffusivity of the active components inside the HA hydrogel three-dimensional network. Such diffusivity depends on the physical–chemical characteristics of the pharmaceutical formulations such as size and ability to form weak bonds with the matrix, and on the pore size of the hydrogel matrix that could generate steric hindrance. In this sense, the fluorescence microscopy is useful too, as it allows for the visualization of the prepared dispersions.

The morphological structure of the complex matrix was observed through SEM analysis, as shown in Figure 5b. It was found that the surface had a relatively homogeneous porous structure. Even though there are differences in the pores’ shape, the morphology of the complex material presented in SEM images proved to have similar pore sizes, with diameters between 2 and 6 μm. The overall aspect is like a honeycomb structure, as can be observed in the included details in Figure 5b, with interconnected pores. The porous network aspect is due to the crosslinking of the HA chains. The obtained structure is similar with the morphology reported by Minaberry et al. [99] where they obtained a HA gel with porous structure applying freeze-drying after the ice-segregation-induced self-assembly procedure. The maintenance at low temperature of the formed HA hydrogel (−20 °C) allows for the formation of a suitable porous-lace-like structure that facilitates the accommodation of other bioactive components. As the content in HA is 3%, a favored release of the antibiotics and MEL is expected.

Figure 5c shows the XRD pattern obtained for the bioactive material. MEL has some sharp peaks at the diffraction angles of 2θ: 17.9°, 24.7°, 25.3° and 26.3°, similar to those identified by Topal et al. [100] considering that MEL has a crystalline structure. There is no sharp peak for the HA, confirming its amorphous nature [101]. Crystalline MZ has intense bands at the diffraction angles of 2θ 12.1° and 23.7°, in close accordance with the data reported by Sabbagh et al. [102]. The presence of T generated peaks at diffraction angles 2θ: 10.5° (low intensity) and 29.8°, which are in good agreement with the data reported in the literature [103]. The XRD results sustain the preservation of the structural characteristics of each compound present in the complex matrix.

The stability of the complex bioactive matrix was assessed through electrochemical determination of zeta potential (ξ), which allows for the assessment of the complex system stability. Zeta potential of the mixture was measured in an aqueous solution (pH = 7.0) at 25 °C. The value obtained for ξ was −36.0 mV ± 1.3. The ξ value indicates a good physical stability of the dispersed complex matrix. Any physical–chemical processes such as ionization or adsorption result in a change in zeta potential. Consequently, a low ξ value suggests the presence of an important amount of charge carriers [104]. The highly negative zeta potential of the complex matrix is due to the incorporation of HA, as the presence of negatively charged HA favors the absorption on the surface.

### 3.2. Cytotoxicity Analysis

**Figure 6:**
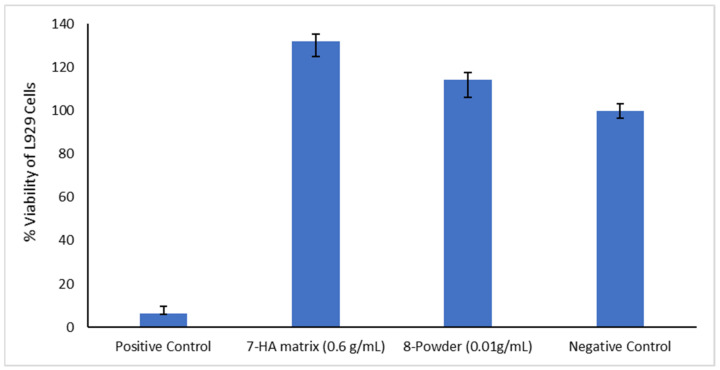
Results of the MTT test.

The results of the cytotoxicity analysis run for L929 line cells are synthetically presented in Figure 6.

The data recorded and presented in Table 1 allow for specific calculations based on an assumed 100% viability for negative control.

According to the cytotoxicity tests, the active samples, either hydrogel or solid, are excellent from the cytotoxicity point of view and highly biocompatible for dental applications. The samples showed significantly higher cell viability compared to the control. These findings indicate cell migration and interaction with the biomaterial. Cell migration and proliferation is increased particularly by HA, the mechanism being due to the release of mitogenic stimulators such as growth factors and the influence of cell-to-cell interaction by forming a pericellular coat, resulting in faster cell proliferation [105].

### 3.3. Microbiological Assessment

The experiments for determining the MIC value led to the results presented in Table 2.

In Figure S5, the evolution of the studied systems under the action of the microbial agents can be seen. MIC for soft paraffin against *Staphylococcus* spp., and *Streptococcus* spp. showed sensitivity at 2500 µg/mL, while the MIC for HA against *Staphylococcus* spp. was 1250 µg/mL, and the strain of *Streptococcus* spp. was resisted at all test concentrations. The MIC for MEL against *Staphylococcus* spp. exhibited sensitivity at 625 µg/mL, and *Streptococcus* spp. highlighted a sensitivity at a concentration of 2500 µg/mL. In the meantime, the MIC for MZ against *Staphylococcus* spp. exhibited sensitivity at 1250 µg/mL and the strain of *Streptococcus* spp. evidenced sensitivity at the concentration of 312.5 µg/mL. As expected, the MIC for T against *Staphylococcus* spp. and *Streptococcus* spp. showed sensitivity at all concentrations tested.

The experimental focus was on the proposed complex mixture’s antibacterial behavior. The antimicrobial behavior of T, MZ, and HA, alone or in combination, is known and well-represented in the literature, as reviewed in the Introduction section. Therefore, the combination of T with HA, or MZ with HA was expected to present the same antibacterial behavior as the individual antibiotics and was not included in the microbiological study. On the other hand, the newly introduced combination of HA and MEL, due to their biological action (MEL improved the quality of alveolar bone, while HA reduces cell proliferation in fibroblasts and lymphocytes, diminishing the inflammatory process at tissues level), was of interest for checking if the antibacterial effects of components are preserved. The obtained results were promising, and upon the further introduction of the antibiotics T and MZ, the antibacterial activity was improved.

The MIC for T, MZ, and soft paraffin (TM paste) against *Staphylococcus* spp. exhibited sensitivity at 1250 µg/mL and for *Streptococcus* spp. the strain showed sensitivity at all tested concentrations. MIC for MEL, HA, and soft paraffin against *Staphylococcus* spp., and *Streptococcus* spp. strains exhibited sensitivity at 2500 µg/mL. The MIC determinations for the solid mixture of MEL and HA against *Staphylococcus* spp. exhibited sensitivity at 625 µg/mL, and for *Streptococcus* spp. the strain showed sensitivity at 2500 µg/mL. The obtained MIC for MEL, MZ, T, and HA matrix against *Staphylococcus* spp. and *Streptococcus* spp. is significant. The bioactive hydrogel matrix showed sensitivity to the tested strains at all concentrations studied. Therefore, it is expected to see an improved local targeted action of the bioactive HA matrix compared to the TM paste. The experimental data highlighted that the dispersion of the antibiotics and MEL in the HA hydrogel matrix does not inhibit the antibacterial activity but sustains it.

### 3.4. pH Influence

As saliva is a complex electrolyte characterized through various values of pH, ranging between 2 and 9 depending on specific health conditions and upon the consumption of products that generate either a low acidity or a high basicity, it would be useful and practical to study the behavior of the complex matrix in an environment mimicking saliva characteristics.

**Figure 7:**
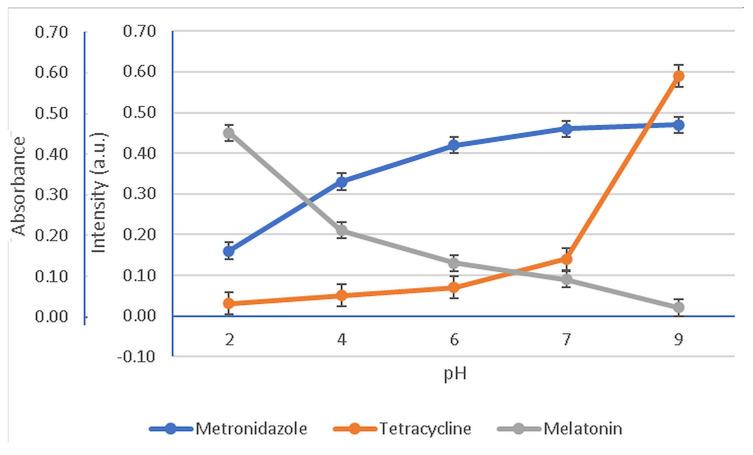
Variation in the normalized absorbance and fluorescence intensities as a function of pH values. Fluorescence readings for MEL at 570 nm (λ_ex_ = 278 nm) and T at 530 nm (λ_ex_ = 335 nm); absorbance readings for MZ at 320 nm.

In Figure 7 are presented the variation tendencies of the absorbances for MZ, specifically the fluorescence intensities for the MEL and T active components dispersed into HA hydrogel membrane.

**Figure 8:**
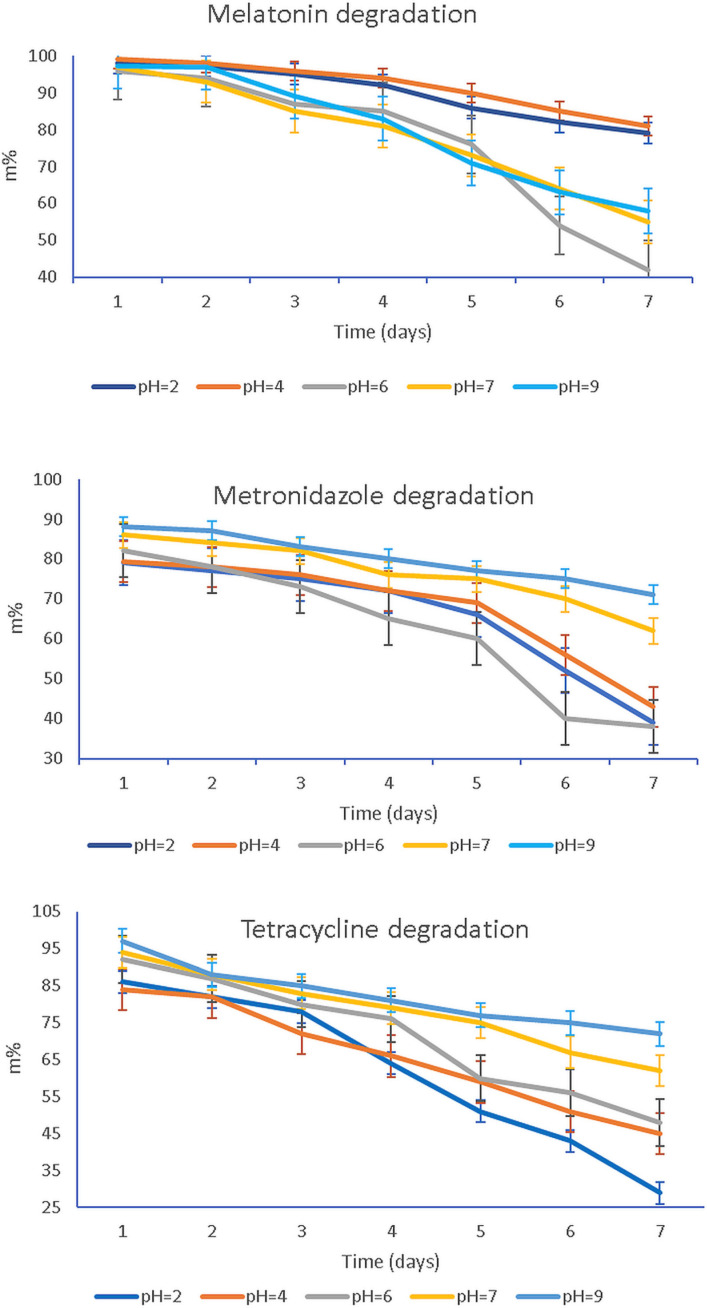
Degradation of the bioactive compounds MEL, MZ, and T as a function of pH values throughout the storage time of 7 days, at 25 °C.

The degradation of MEL, T, and MZ under storage standard conditions for one week as a function of the solution pH is presented in Figure 8.

The plots in Figure 8 report the remaining mass of active compounds MEL, T, MZ, namely, the m% that was calculated according to the equation:

(8) $$m\%=\frac{m_{t}}{m_{o}}\cdot 100$$

where m_t_ stands for active compounds at time t, while m_0_ is the initial mass of the same considered compound.

To evaluate the effect of pH on the stability of MEL, T, and MZ, results were compared to their concentration in electrolytes with pH values of 2, 4, 6, 7, and 9, as illustrated in Figure 8. The active compounds’ contents in all pH solutions decreased with increasing storage time, and degradation of all studied formulations in pH solutions demonstrated two different comportments depending on the pH value, namely, a slow or a dramatic degradation in time.

### 3.5. Release Profiles

We recorded experimental data for the three bioactive formulations, MEL, MZ, and T, following the release against the concentration of analyte measured in the diffusion cell at different times between 12 and 168 h covering a one-week timeframe.

**Figure 9:**
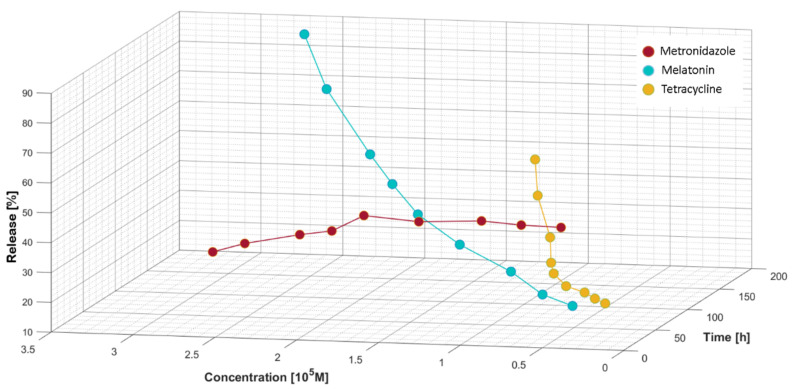
Release ratio (%) versus concentration of bioactive components and time.

The release profile as release ratio (%) for MEL, T, and MZ can be followed in Figure 9. For MEL, from a concentration of 1.80 × 10^−5^ mol/L we observe a faster release from the hydrogel matrix that could be assimilated with a burst region. The release profile was calculated according to:

(9) $$R\%=\frac{A_{0}-A_{t}}{A_{0}}\cdot 100=\frac{c_{0}-c_{t}}{c_{0}}\cdot 100$$

where A_0_ is the absorbance recorded for the initial concentration c_0_ (mol/L), and A_t_ is the absorbance recorded at a certain t when the component concentration is c_t_ (mol/L).

The absorbance variation for MZ against the concentration highlights the initial slow release of the antibiotic. The releasing profile recorded for MZ, shown in Figure 9, indicates a continuous yet slow increase in its delivery from the moment of applying the treatment mixture containing the bioactive compounds. In the burst phase, up to 2.40 × 10^−5^ mol/L MZ is released in a quasi-constant manner, followed by a decrease in its delivery. For T, a similar release profile to MEL was recorded. Up to a concentration of 0.90 × 10^−5^ mol/L for T, a quasi-constant release was observed, followed by a burst region. The highest release values decreased in the following order: MEL > T > MZ.

Improving the transport mechanism across the HA matrix of the active formulations and practical design of a proper release system is possible through a correct interpretation and understanding of the release kinetics. The diffusion equilibrium information is a valuable tool for modeling the releasing process through the used HA hydrogel membranes. Using the above-presented experimental data, various models were applied for each active compound of the topical treatment to assess the best-fitting kinetic model.

**Figure 10:**
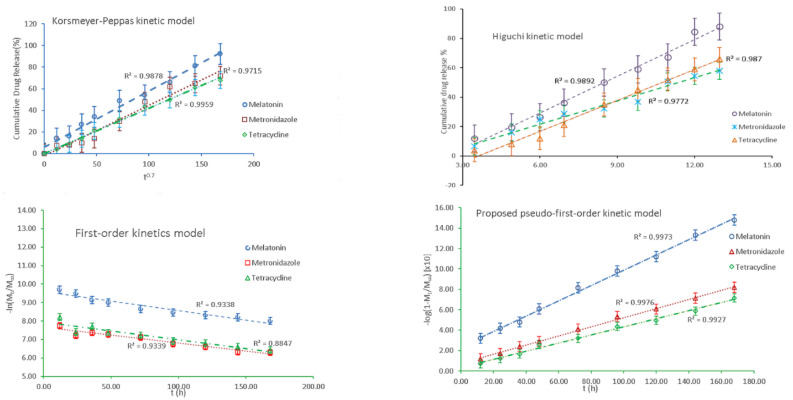
The kinetic models describing the release mechanism for each active compound.

Figure 10 presents the processed experimental data applying the models that appropriately describe the specific mechanism for each analyte: MEL, MZ, and T. The Higuchi and Korsmeyer–Peppas kinetic equations, that were applied to model the behavior of the MZ across the HA matrix, provide evidence that the release processes occur in a similar manner to that of T, although it was expected that MZ would diffuse from the HA matrix much faster and easier than T which has a much larger volume.

The models also apply to the release mechanism from heterogeneous surfaces. However, it should be mentioned that such modeling kinetics applies only to the intermediate range of released concentrations, reflecting an empirical approach.

Unfortunately, a unitary model was not identified that could be applied to all the analytes considered. Some other models could likely fit better the recorded experimental data. Thus, we proposed an alternative kinetic model involving a possible pseudo-first-order kinetic model that could be expressed as:

(10) $$\log\left(1-\frac{M_{t}}{M_{\infty }}\right)=-\frac{k_{1}}{2.303}\cdot t$$

where k_1_ is the pseudo-first-order rate constant, M_t_ is the total quantity of drug released analyte after a particular time t, M_∞_ is the total quantity of dispersed drug into the matrix that should be released, and t stands for the release time. The experimental data represented according to the proposed model for pseudo-first-order kinetics are shown in the diagram from Figure 10.

Table 3 summarizes the corresponding kinetic model parameters, with their correlation coefficients (R^2^) and the related standard errors for each analyte. The data recorded in Table 3 were selected based on the best correlation coefficient and the related standard error. For MEL, the proposed kinetic models resulted equally in a best fit of the experimental data, R^2^ = 0.9976. Meanwhile, for MZ and T, the pseudo-first-order model best fitted the data obtained. The standard deviation errors for the Higuchi model cause substantial fluctuations in R^2^, and thus, the parameters may induce deviation.

Considering the better R^2^ values recorded for MEL and MZ when applying the Higuchi model, known as a particular situation of the Korsmeyer–Peppas model [106], it could be concluded that the release of the active formulations is not due to the HA hydrogel swelling. It would be expected to have a releasing mechanism predominantly through diffusion. As for T, a better correlation coefficient is recorded for the Peppas diffusion exponent n equal to 0.7, suggesting that T is released following the swelling of HA matrix. In such a case, the drugs dispersed into the HA hydrogel do not entirely obey Fick-type diffusion, signifying that the MEL, MZ, and in a lower extent T, are facing a limited diffusion through HA structural network, although their dimensions are smaller than the HA mesh. In the case of larger drug molecule such as T, it is expected that its release would be faster upon the deformation of HA’s three-dimensional porous structure due to the swelling when in contact with saliva. The values obtained for the Korsmeyer–Peppas model when applied for T could be considered supportive of such releasing mechanism. The proposed pseudo-first-order model could assess more accurately the behavior of the releasing profile of the bioactive compounds, taking into account the amount of the remaining drugs in the HA membrane. It is very likely that the simultaneous presence of the active formulations generates reciprocal hindrance and competitive diffusion. Furthermore, the difference in the volume of each component should be also considered.

## 4. Discussion

In view of the future usage of the proposed topical formulation for the treatment of periodontal wounds, it is obviously necessary to determine how the bioactive components against the pH value react. Additionally, by correlating the behavior of the drugs in the complex combination, the optimal value of the working pH can be established to determine how the components are released into the saliva. When considering the drug release from a hydrogel matrix such as HA, we should consider the pH influence on the solubility of the components or on the possible interactions between the hydrogel matrix and the dispersed drug. Such influences may impact the release profiles. Thus, controlling pH with respect to the relevant value provides hints for an appropriate release during in vivo experiments.

The study of the pH influence on the bioactive components’ stability in time evidenced either a slow or quick and significant degradation. Thus, a slower degradation rate during storage was observed for MEL at pH = 2 and pH = 4. Meanwhile, the solutions characterized by pH value 6, 7, and 9 degraded significantly with storage time. The slow degradation in acidic environment suggests that MEL is more stable at low pH value. The increasing instability of MEL when the solutions turn from neutral to basic could be assigned to the indole group behavior, as it could suffer a deprotonation of the amide function. In contrast, for MZ an abrupt degradation in the acidic solution, pH < 6, and a slow degradation when the pH is neutral towards basic, pH = 7–9, were recorded. The third studied compound, T, showed a similar behavior to MZ, changing from fast degradation in acidic media, pH = 2–6, to a slow one for pH = 7–9. Studying the HA complex matrix behavior against pH, Figure 7, it was observed that MZ and T were released at higher active concentrations than MEL when the pH was increased from 6.75, specific for patients with periodontitis, to a pH of 7.10, characterizing healthy patients. Although there is not a consistent component of MEL, T, and MZ regarding their preservation in time as a function of pH, pH = 7.0 was chosen as the working pH for further release studies of the drugs from the HA hydrogel membrane. The rationale of the working pH = 7 is based on the fact that at this value all active components presented a slow degradation in time, even if after 7 days for MEL there was a more pronounced degradation to 56% of its initial mass compared with MZ (62%) and T (64%).

The proposed kinetic models consider the possibility that the release mechanism governing the hydrogel system with three active components could be supported by a nonlinear kinetic model where both diffusional and swelling components could be involved. The experimental data are reflected in cumulative release diagrams where the specific behavior for each component is visualized. Although a common release profile supposes a burst phase when the drug is released quickly and then a slow in the release rate that generates the formation of a plateau, the experimental data obtained for our mixture do not comply with such a release profile. Only MZ presented a fast and significant release in the first 48 h, followed by a slower release and the recording of a plateau—Figure 8. Both MEL and T presented a continuous release from the HA matrix, with a clear increasing trend for MEL. If for T up to 0.90 × 10^−5^ mol/L, the release values showed a limited increase describing a plateau, for MEL the release profile had no difference between a burst and plateau region, although from 1.80 × 10^−5^ mol/L the increasing trend was more obvious. Regarding the behavior of T, we observed an initially short plateau for the release profile which was followed by an increase that is in reversed order, as expected. This could be explained by the difficulty to be released quickly through diffusion from the HA matrix, with a certain swelling of the HA hydrogel being necessary in order to have a higher release of T. Comparing the recorded values for the applied models, the results obtained applying the proposed pseudo-first-order kinetic model better suit the recorded experimental data.

In this preliminary study, we followed up the variation in the three analytical vectors, namely the concentration of T, MZ, and MEL, when a HA matrix membrane is introduced into the system. The general release profile of the studied system does not comply with the commonly applied models and with accepted scenarios for the drug release from hydrogels. This aspect is due to the complexity of the studied mixture, as there were three bioactive compounds dispersed into the HA hydrogel membrane, not only one. Additionally, it should be considered the physical interaction between MEL, T and MZ with the HA hydrogel forms weak H-H bonds. The experience in developing a guided bone barrier membrane [107] and the results recorded during this study encourage us to couple the new topical treatment proposed for periodontal disease with the application of a HA hydrogel membrane for controlling the active formulation’s release.

HA hydrogel membrane as a potential carrier for active compounds used for the proposed topical treatment of periodontal disease is due to the biocompatibility and easily tunable characteristics of different HA derivatives. Further studies with an extended pH range and characterizing the varied health status of different patients are required to obtain a tunable membrane for an optimal release of MEL and antibiotics in clinical applications.

## 5. Conclusions

All the active substances tested alone and combined had a biological response to the strains of *Staphylococcus* spp. and *Streptococcus* spp., expressed by sensitivity/resistance to the specific action mechanism of the bacteria tested. The obtained results during the microbiological assessment indicate that the bioactive complex mixture (MEL, HA, MZ, T) can be a valuable and an economic resource for use in the treatment of periodontal disease. Additionally, it may have potential in clinical applications as a disinfectant for various surgical and orthodontic appliances, and maintaining oral hygiene.

Regarding the pH influence on the stability of MEL, MZ, and T in time, we observed their degradation. Upon increasing pH within the physiological range, MEL was released at lower active concentrations compared to MZ and T. Thus, at pH characterizing healthy patients (pH = 7.10), the degradation of T or MZ was significantly slower, despite MEL which is stable in acidic media. Based on these observations, pH = 7.0 was chosen as the working pH for MEL, MZ and T release profiles from the HA membrane.

The preliminary study on the controlled delivery of the active compounds through the HA membrane allowed the modeling through various kinetic models. As there was not a unitary model for all active compounds, we proposed an alternative kinetic model. The proposed pseudo-first-order model adequately described the release of all bioactive compounds.

The release profiles of the bioactive analytes from the HA matrix membrane showed specific characteristics: MZ faced a burst phase followed by a continuous release decrease and not by a plateau as expected; MEL from a concentration of 1.80 × 10^−5^ mol/L recorded a quick release in time; and T from 0.901 × 10^−5^ mol/L presented similar behavior to MEL, after having a limited plateau of release values.

Further investigations are needed to clarify the dependence of the release behavior on the pH variation and temperature. Nevertheless, the results are encouraging for the usage of the HA matrix as a releasing vehicle for the active components of the proposed topical treatment.

## 6. Patents

“Biocompatible paste material useful for topical treatment of slowly progressive chronic marginal periodontitis, comprises T, MZ, MEL, HA, white Vaseline” Patent pending no RO133752-A0; Derwent Manual Code(s): B02-T; B04-B01C3; B04-C02E4; B06-D01; B07-D09; B12-M02A; B12-M12B; B14-N06B; B14-S09; B14-S18.

## Figures and Tables

**Table 1:** Data for MTT test.

Material	Mean Absorbance	% Viability
7-HA matrix (0.6 g/mL)	2.443 ± 0.070	132.09
8-Powder (0.01 g/mL)	2.112 ± 0.081	114.19
Negative control	1.850 ± 0.033	100.00
Positive control	0.117 ± 0.005	6.34

**Table 2:** Antimicrobial activity of HA, MEL, MZ, T, soft paraffin, and mixtures against *Staphylococcus* spp. and *Streptococcus* spp.

No.	Sample/ AS Concentration µg/mL	Microbial Strains									
		*Staphylococcus* spp.					*Streptococcus* spp.				
		2500	1250	625	312.5	156.25	2500	1250	625	312.5	156.25
1	T, MZ, Soft paraffin	S	S	R	R	R	S	S	S	S	S
2	MEL, HA, Soft paraffin	S	R	R	R	R	S	R	R	R	R
3	MEL, HA—solid sample	S	S	S	R	R	S	R	R	R	R
4	MZ, T, MEL, HA matrix	S	S	S	S	R	S	S	S	S	S
5	Soft paraffin	S	R	R	R	R	S	R	R	R	R
6	HA	S	S	R	R	R	R	R	R	R	R
7	MEL	S	S	S	R	R	S	R	R	R	R
8	MZ	S	S	R	R	R	S	S	S	R	R
9	T	S	S	S	S	S	S	S	S	S	S
10	Control	+	+	+	+	+	+	+	+	+	+

AS—active substance; S—sensitivity; R—resistance.

**Table 3:** The kinetic constants obtained by linear fitting for the studied analytes.

Kinetic Model	Parameter	Analyte		
		MEL	MZ	T
Higuchi	k	3.79 × 10^−4^ ± 0.84	2.26 × 10^−4^ ± 0.72	1.49 × 10^−4^ ± 0.21
	R^2^	0.9892	0.9870	0.9772
Korsmeyer–Peppas	k	2.37 × 10^−2^ ± 0.14	4.36 × 10^−2^ ± 0.51	7.27 × 10^−2^ ± 0.77
	R^2^	0.9843	0.9787	0.9793
Pseudo-first-order	k	1.62 × 10^−3^ ± 0.35	0.88 × 10^−3^ ± 0.47	0.73 × 10^−3^ ± 0.31
	R^2^	0.9976	0.9973	0.9927
First-order	k	2.06 × 10^−3^ ± 0.49	0.66 × 10^−3^ ± 0.55	0.60 × 10^−3^ ± 0.14
	R^2^	0.9338	0.8847	0.9339

## Supplementary Materials

The following supporting information can be downloaded at: https://www.mdpi.com/article/10.3390/membranes12030303/s1, Figure S1: Thermal analysis; Figure S2: FTIR spectra; Figure S3: UV Spectrum for HA hydrogel form; Figure S4: Fluorescence spectra (detailed) for each component: individually and in complex mixture; Figure S5: The action of the microbial agents on the systems based on the bioactive formulation.

## References

[1] Seong Y.-J., Lin G., Kim B.J., Kim H.-E., Kim S., Jeong S.-H. (2019). Hyaluronic Acid-Based Hybrid Hydrogel Microspheres with Enhanced Structural Stability and High Injectability. ACS Omega.

[2] Sun B., Zhang M., Shen J., He Z., Fatehi P., Ni Y. (2019). Applications of cellulose-based materials in sustained drug delivery systems. Curr. Med. Chem..

[3] Chatterjee S., Hui P.C.-L., Siu W.S., Kan C.-W., Leung P.-C., Wanxue C., Chiou J.-C. (2021). Influence of pH-responsive compounds synthesized from chitosan and hyaluronic acid on dual-responsive (pH/temperature) hydrogel drug delivery systems of Cortex Moutan. Int. J. Biol. Macromol..

[4] Zhu J., Tang X., Jia Y., Ho C.T., Huang Q. (2020). Applications and delivery mechanisms of hyaluronic acid used for topical/transdermal delivery—A review. Int. J. Pharm..

[5] Kim H.S., Yang J., Kim K., Shin U.S. (2019). Biodegradable and injectable hydrogels as an immunosuppressive drug delivery system. Mater. Sci. Eng. C.

[6] Verhoef J.J.F., Anchordoquy T.J. (2013). Questioning the use of PEGylation for drug delivery. Drug Deliv. Transl. Res..

[7] Elashiry M., Meghil M.M., Arce R.M., Cutler C.W. (2019). From manual periodontal probing to digital 3-D imaging to endoscopic capillaroscopy: Recent advances in periodontal disease diagnosis. J. Periodontal. Res..

[8] Marsh P.D., Zaura E. (2017). Dental biofilm: Ecological interactions in health and disease. J. Clin. Periodontol..

[9] Abou Neel E.A., Aljabo A., Strange A., Ibrahim S., Coathup M., Young A.M., Bozec L., Mudera V. (2016). Demineralization-remineralization dynamics in teeth and bone. Int. J. Nanomed..

[10] Jepsen K., Jepsen S. (2016). Antibiotics/antimicrobials: Systemic and local administration in the therapy of mild to moderately advanced periodontitis. Periodontology 2000.

[11] Pilloni A., Zeza B., Kuis D., Vrazic D., Domic T., Olszewska-Czyz I., Popova C., Kotsilkov K., Firkova E., Dermendzieva Y. (2021). Treatment of Residual Periodontal Pockets Using a Hyaluronic Acid-Based Gel: A 12 Month Multicenter Randomized Triple-Blinded Clinical Trial. Antibiotics.

[12] Cho Y.D., Kim K.H., Lee Y.M., Ku Y., Seol Y.J. (2021). Periodontal Wound Healing and Tissue Regeneration: A Narrative Review. Pharmaceuticals.

[13] Henskens Y.M., van der Weijden F.A., van den Keijbus P.A., Veerman E.C., Timmerman M.F., van der Velden U., Amerongen A.V. (1996). Effect of periodontal treatment on the protein composition of whole and parotid saliva. J. Periodontol..

[14] Galgut P.N. (2001). The relevance of pH to gingivitis and periodontitis. J. Int. Acad. Periodontol..

[15] Muñoz-Carrillo J., Hernández-Reyes V.E., García-Huerta O.E., Chávez-Ruvalcaba F., Chávez-Ruvalcaba M.I., Chávez-Ruvalcaba K.M., Díaz-Alfaro L., Yussif N.M.A. (2019). Pathogenesis of Periodontal Disease. Periodontal Disease: Diagnostic and Adjunctive Non-surgical Considerations.

[16] Dahiya P., Kamal R. (2013). Hyaluronic Acid: A boon in periodontal therapy. N. Am. J. Med. Sci..

[17] Bonito A.J., Lux L., Lohr K.N. (2005). Impact of local adjuncts to scaling and root planing in periodontal disease therapy: A systematic review. J. Periodontol..

[18] Goldberg M. (2020). Antibiotics and Antibacterial Medications for Endodontic Treatments. JSM Dent..

[19] Passarelli P.C., Netti A., Lopez M.A., Giaquinto E.F., De Rosa G., Aureli G., Bodnarenko A., Papi P., Starzyńska A., Pompa G. (2021). Local/Topical Antibiotics for Peri-Implantitis Treatment: A Systematic Review. Antibiotics.

[20] Junmahasathien T., Panraksa P., Protiarn P., Hormdee D., Noisombut R., Kantrong N., Jantrawut P. (2018). Preparation and Evaluation of Metronidazole-Loaded Pectin Films for Potentially Targeting a Microbial Infection Associated with Periodontal Disease. Polymers.

[21] Fu Y., Rubio A.H., Gscheider C., du Teil Espina M., del Carmen Flores-Vallejo R., van Dijl J.M., Gabarrini G. (2021). Oral and Dental Infections: Bacteria. Ref. Modul. Biomed. Sci..

[22] Loesche W.J., Grossman N.S. (2001). Periodontal Disease as a Specific, albeit Chronic, Infection: Diagnosis and Treatment. Clin. Microbiol. Rev..

[23] Heitz-Mayfield L.J.A. (2009). Systemic antibiotics in periodontal therapy. Aust. Dent. J..

[24] Matesanz-Pérez P., García-Gargallo M., Figuero E., Bascones-Martínez A., Sanz M., Herrera D. (2013). A systematic review on the effects of local antimicrobials as adjuncts to subgingival debridement, compared with subgingival debridement alone, in the treatment of chronic periodontitis. J. Clin. Periodontol..

[25] Lai C.K.C., Ng R.W.Y., Leung S.S.Y., Hui M., Ip M. (2022). Overcoming the rising incidence and evolving mechanisms of antibiotic resistance by novel drug delivery approaches—An overview. Adv. Drug Deliv. Rev..

[26] Kumar M., Sarma D.K., Shubham S., Kumawat M., Verma V., Nina P.B., Jp D., Kumar S., Singh B., Tiwari R.R. (2021). Futuristic Non-antibiotic Therapies to Combat Antibiotic Resistance: A Review. Front. Microbiol..

[27] Chandler C.I.R. (2019). Current accounts of antimicrobial resistance: Stabilisation, individualisation and antibiotics as infrastructure. Palgrave Commun..

[28] Vanić Ž., Jøraholmen M.W., Škalko-Basnet N. (2021). Nanomedicines for the topical treatment of vulvovaginal infections: Addressing the challenges of antimicrobial resistance. Adv. Drug Deliv. Rev..

[29] Cristache C.M., Totu E.E., Burlibasa M., Tanase G., Iorgulescu G., Burlibasa L. (2020). Preliminary Study on Genotoxicity Assessment of an Innovative Topical Treatment for Periodontal Disease. Rev. Chim..

[30] Mhaske N., Sheker A., Marawar P., Mote N. (2010). Evaluation of melatonin levels in saliva in periodontal health and disease: A clinico-biochemical study. J. Int. Clin. Dent. Res. Organ..

[31] Marawar A., Marawar P., Nandal D.H., Kunkulol R., Narwane S. (2019). Evaluation of Effect of Melatonin on Hematological Parameters in Patients of Periodontitis. Int. J. Clin. Biomed. Res..

[32] Balaji T.M., Varadarajan S., Jagannathan R., Mahendra J., Fageeh H.I., Fageeh H.N., Mushtaq S., Baeshen H.A., Bhandi S., Gupta A.A. (2021). Melatonin as a topical/systemic formulation for the management of periodontitis: A systematic review. Materials.

[33] Hardeland R. (2018). Melatonin and inflammation—Story of a double-edged blade. J. Pineal Res..

[34] Nabavi S.M., Nabavi S.F., Sureda A., Xiao J., Dehpour A.R., Shirooie S., Silva A.S., Baldi A., Khan H., Daglia M. (2019). Anti-inflammatory effects of Melatonin: A mechanistic review. Crit. Rev. Food Sci. Nutr..

[35] El-Sharkawy H., Elmeadawy S., Elshinnawi U., Anees M. (2019). Is dietary melatonin supplementation a viable adjunctive therapy for chronic periodontitis?—A randomized controlled clinical trial. J. Periodontal Res..

[36] Castro M.M.L., Duarte N.N., Nascimento P.C., Magno M.B., Fagundes N.C.F., Flores-Mir C., Monteiro M.C., Rösing C.K., Maia L.C., Lima R.R. (2019). Antioxidants as Adjuvants in Periodontitis Treatment: A Systematic Review and Meta-Analysis. Oxid. Med. Cell. Longev..

[37] Bazyar H., Gholinezhad H., Moradi L., Salehi P., Abadi F., Ravanbakhsh M., Zare Javid A. (2019). The effects of melatonin supplementation in adjunct with non-surgical periodontal therapy on periodontal status, serum melatonin and inflammatory markers in type 2 diabetes mellitus patients with chronic periodontitis: A double-blind, placebo-controlled t. Inflammopharmacology.

[38] Balaji T.M., Vasanthi H.R., Rao S.R. (2015). Gingival, plasma and salivary levels of melatonin in periodontally healthy individuals and chronic periodontitis patients: A pilot study. J. Clin. Diagnostic Res..

[39] Balaji T.M., Varadarajan S., Jagannathan R., Gupta A.A., Raj A.T., Patil S., Fageeh H.I., Fageeh H.N. (2020). Melatonin levels in periodontitis vs. the healthy state: A systematic review and meta-analysis. Oral Dis..

[40] Tuncay Tanrıverdi S., Cheaburu-Yilmaz C.N., Carbone S., Özer Ö., Tuncay Tanrıverdi S., Cheaburu-Yilmaz C.N., Carbone S.O. (2018). Preparation and in vitro evaluation of melatonin-loaded HA/PVA gel formulations. Pharm. Dev. Technol..

[41] Kovacic P., Somanathan R. (2014). Melatonin and Circadian Rhythm: Aging, Cancer, and Mechanism. Open J. Prev. Med..

[42] Konečná B., Chobodová P., Janko J., Baňasová L., Bábíčková J., Celec P., Tóthová Ľ. (2021). The Effect of Melatonin on Periodontitis. Int. J. Mol. Sci..

[43] Mayo J.C., Tan D.X., Sainz R.M., Lopez-Burillo S., Reiter R.J. (2003). Oxidative damage to catalase induced by peroxyl radicals: Functional protection by melatonin and other antioxidants. Free Radic. Res..

[44] Ma X., Idle J.R., Krausz K.W., Gonzalez F.J. (2005). Metabolism of melatonin by human cytochromes P450. Drug Metab. Dispos..

[45] Zhao D., Yu Y., Shen Y., Liu Q., Zhao Z., Sharma R., Reiter R.J. (2019). Melatonin Synthesis and Function: Evolutionary History in Animals and Plants. Front. Endocrinol..

[46] Carpentieri A.R., Oliva C., Díez-Noguera A., Cambras T., Carpentieri A.R., Oliva C., Díez-Noguera A.C. (2015). Melatonin administration modifies circadian motor activity under constant light depending on the lighting conditions during suckling. Chronobiol. Int..

[47] Kim T.K., Kleszczynśki K., Janjetovic Z., Sweatman T., Lin Z., Li W., Reiter R.J., Fischer T.W., Slominski A.T. (2013). Metabolism of melatonin and biological activity of intermediates of melatoninergic pathway in human skin cells. FASEB J..

[48] Ressmeyer A.R., Mayo J.C., Zelosko V., Sáinz R.M., Tan D.X., Poeggeler B., Antolín I., Zsizsik B.K., Reiter R.J., Hardeland R. (2003). Antioxidant properties of the melatonin metabolite N1-acetyl-5-methoxykynuramine (AMK): Scavenging of free radicals and prevention of protein destruction. Redox Rep..

[49] Cagnoli C.M., Atabay C., Kharlamova E., Manev H. (1995). Melatonin protects neurons from singlet oxygen-induced apoptosis. J. Pineal Res..

[50] Escames G., Guerrero J.M., Reiter R.J., Garcia J.J., Munoz-Hoyos A., Ortiz G.G., Oh C.S. (1997). Melatonin and vitamin E limit nitric oxide-induced lipid peroxidation in rat brain homogenates. Neurosci. Lett..

[51] Rodriguez C., Mayo J.C., Sainz R.M., Antolín I., Herrera F., Martín V., Reiter R.J. (2004). Regulation of antioxidant enzymes: A significant role for melatonin. J. Pineal Res..

[52] Wang Y., Andrukhov O., Rausch-Fan X. (2017). Oxidative stress and antioxidant system in periodontitis. Front. Physiol..

[53] Reiter R.J., Mayo J.C., Tan D.X., Sainz R.M., Alatorre-Jimenez M., Qin L. (2016). Melatonin as an antioxidant: Under promises but over delivers. J. Pineal Res..

[54] Galano A., Tan D.X., Reiter R.J. (2011). Melatonin as a natural ally against oxidative stress: A physicochemical examination. J. Pineal Res..

[55] Amstrup A.K., Sikjaer T., Mosekilde L., Rejnmark L. (2013). Melatonin and the skeleton. Osteoporos. Int..

[56] Olszewska-Czyz I., Kralik K., Prpic J. (2021). Biomolecules in dental applications: Randomized, controlled clinical trial evaluating the influence of hyaluronic acid adjunctive therapy on clinical parameters of moderate periodontitis. Biomolecules.

[57] Jiang D., Liang J., Noble P.W. (2011). Hyaluronan as an Immune Regulator in Human Diseases. Physiol. Rev..

[58] David-Raoudi M., Tranchepain F., Deschrevel B., Vincent J.C., Bogdanowicz P., Boumediene K., Pujol J.P. (2008). Differential effects of hyaluronan and its fragments on fibroblasts: Relation to wound healing. Wound Repair Regen..

[59] Litwiniuk M., Krejner A., Grzela T. (2016). Hyaluronic Acid in Inflammation and Tissue Regeneration. Wounds A Compend. Clin. Res. Pract..

[60] Gall Y. (2010). Hyaluronic acid: Structure, metabolism and implication in cicatrisation. Ann. Dermatol. Venereol..

[61] Sierra-Sánchez Á., Fernández-González A., Lizana-Moreno A., Espinosa-Ibáñez O., Martinez-Lopez A., Guerrero-Calvo J., Fernández-Porcel N., Ruiz-García A., Ordóñez-Luque A., Carriel V. (2020). Hyaluronic acid biomaterial for human tissue-engineered skin substitutes: Preclinical comparative in vivo study of wound healing. J. Eur. Acad. Dermatol. Venereol..

[62] Asparuhova M.B., Chappuis V., Stähli A., Buser D., Sculean A. (2020). Role of hyaluronan in regulating self-renewal and osteogenic differentiation of mesenchymal stromal cells and pre-osteoblasts. Clin. Oral Investig..

[63] Valachová K., Šoltés L. (2021). Assessment of the Substance Antioxidative Profile by Hyaluronan, Cu(II) and Ascorbate. Pharmaceutics.

[64] Pagnacco A., Vangelisti R., Erra C., Poma A. (1997). Double-blind clinical trial vs. placebo of a new sodium-hyaluronate-based gingival gel. Attual. Ter. Internazionale.

[65] Gontiya G., Galgali S.R. (2012). Effect of hyaluronan on periodontitis: A clinical and histological study. J. Indian Soc. Periodontol..

[66] Eick S., Renatus A., Heinicke M., Pfister W., Stratul S.-I.S., Jentsch H. (2013). Hyaluronic Acid as an Adjunct After Scaling and Root Planing: A Prospective Randomized Clinical Trial. J. Periodontol..

[67] Bansal J., Kedige S.D., Anand S. (2010). Hyaluronic acid: A promising mediator for periodontal regeneration. Indian J. Dent. Res..

[68] Bayer I.S. (2020). Hyaluronic Acid and Controlled Release: A Review. Molecules.

[69] Ma X., Liu S., Tang H., Yang R., Chi B., Ye Z. (2018). In situ photocrosslinked hyaluronic acid and poly (γ-glutamic acid) hydrogels as injectable drug carriers for load-bearing tissue application. J. Biomater. Sci. Polym. Ed..

[70] Malizos K., Blauth M., Danita A., Capuano N., Mezzoprete R., Logoluso N., Drago L., Romanò C.L. (2017). Fast-resorbable antibiotic-loaded hydrogel coating to reduce post-surgical infection after internal osteosynthesis: A multicenter randomized controlled trial. J. Orthop. Traumatol..

[71] Shoham N., Sasson A.L., Lin F.H., Benayahu D., Haj-Ali R., Gefen A. (2013). The mechanics of hyaluronic acid/adipic acid dihydrazide hydrogel: Towards developing a vessel for delivery of preadipocytes to native tissues. J. Mech. Behav. Biomed. Mater..

[72] Oliva F., Marsilio E., Asparago G., Frizziero A., Berardi A.C., Maffulli N. (2021). The impact of hyaluronic acid on tendon physiology and its clinical application in tendinopathies. Cells.

[73] Hahn S.K., Oh E.J., Miyamoto H., Shimobouji T. (2006). Sustained release formulation of erythropoietin using hyaluronic acid hydrogels crosslinked by Michael addition. Int. J. Pharm..

[74] Hsiao M.Y., Lin A.C., Liao W.H., Wang T.G., Hsu C.H., Chen W.S., Lin F.H. (2019). Drug-loaded hyaluronic acid hydrogel as a sustained-release regimen with dual effects in early intervention of tendinopathy. Sci. Rep..

[75] Cristache C.M., Totu E.E., Petre D., Buga R., Cristache G., Totu T. (2018). Melatonin and Hyaluronic Acid Mixture as a Possible Therapeutic Agent in Dental Medicine. Rev. Chim. Buchar. Orig. Ed..

[76] Cristache C.M., Totu E.E., Cristache G., Nechifor A.C., Pintilie I.I. (2019). Melatonin and Hyaluronic Acid in Periodontal Disease. Rev. Chim..

[77] Totu E.E., Cristache C.M., Buga R., Dumitru F., Totu T. (2019). A Card Double Face: Compounds’ Functionality and Synergy of a Topical Treatment Proposed for Oral Health Improvement in Periodontal Disease. Rev. Chim..

[78] Cristache C.M., Totu E.E., Tanase G., Nechifor A.C., Petre D., Burlibasa M. (2019). Innovative Complex Formulation as Topical Treatment for Oral Health Improvment in Periodontal Disease. Rev. Chim..

[79] National Center for Biotechnology Information PubChem Compound Summary for CID 896, Melatonin. https://pubchem.ncbi.nlm.nih.gov/compound/Melatonin.

[80] National Center for Biotechnology Information PubChem Compound Summary for CID 4173, Metronidazole. https://pubchem.ncbi.nlm.nih.gov/compound/Metronidazole.

[81] National Center for Biotechnology Information PubChem Compound Summary for CID 54675776, Tetracycline. https://pubchem.ncbi.nlm.nih.gov/compound/Tetracycline.

[82] Van Meerloo J., Kaspers G.J.L., Cloos J. (2011). Cell sensitivity assays: The MTT assay. Methods Mol. Biol..

[83] Othersen O.G., Beierlein F., Lanig H., Clark T. (2003). Conformations and tautomers of tetracycline. J. Phys. Chem. B.

[84] Stando K., Kasprzyk P., Felis E., Bajkacz S. (2021). Heterogeneous Photocatalysis of Metronidazole in Aquatic Samples. Molecules.

[85] Bickel M., Munoz J.L., Giovannini P. (1985). Acid-base properties of human gingival crevicular fluid. J. Dent. Res..

[86] Pimenta A.F.R., Serro A.P., Colaço R., Chauhan A. (2019). Optimization of intraocular lens hydrogels for dual drug release: Experimentation and modelling. Eur. J. Pharm. Biopharm..

[87] Toffoletto N., Saramago B., Serro A.P. (2020). Therapeutic Ophthalmic Lenses: A Review. Pharmaceutics.

[88] Brown M.B., Jones S.A. (2005). Hyaluronic acid: A unique topical vehicle for the localized delivery of drugs to the skin. J. Eur. Acad. Dermatol. Venereol..

[89] Korsmeyer R.W., Peppas N.A. (1981). Effect of the morphology of hydrophilic polymeric matrices on the diffusion and release of water soluble drugs. J. Memb. Sci..

[90] Kodavaty J., Deshpande A.P. (2020). Evaluation of solute diffusion and polymer relaxation in cross-linked hyaluronic acid hydrogels: Experimental measurement and relaxation modeling. Polym. Bull..

[91] Yotsuyanagi T., Higuchi W.I., Ghanem A.H. (1973). Theoretical treatment of diffusional transport into and through an oil-water emulsion with an interfacial barrier at the oil-water interface. J. Pharm. Sci..

[92] Mircioiu C., Voicu V., Anuta V., Tudose A., Celia C., Paolino D., Fresta M., Sandulovici R., Mircioiu I. (2019). Mathematical Modeling of Release Kinetics from Supramolecular Drug Delivery Systems. Pharmaceutics.

[93] Vigata M., Meinert C., Hutmacher D.W., Bock N. (2020). Hydrogels as Drug Delivery Systems: A Review of Current Characterization and Evaluation Techniques. Pharmaceutics.

[94] Kim S.J., Shin S.R., Lee S.M., Kim I.Y., Kim S.I. (2003). Thermal characteristics of polyelectrolyte complexes composed of chitosan and hyaluronic acid. J. Macromol. Sci. Pure Appl. Chem..

[95] Fukumoto K., Yoshizawa M., Ohno H. (2005). Room temperature ionic liquids from 20 natural amino acids. J. Am. Chem. Soc..

[96] Singh G., Abbas J.M., Dogra S.D., Sachdeva R., Rai B., Tripathi S.K., Prakash S., Sathe V., Saini G.S.S. (2014). Vibrational and electronic spectroscopic studies of melatonin. Spectrochim. Acta A Mol. Biomol. Spectrosc..

[97] Zafra-Roldán A., Corona-Avendaño S., Montes-Sánchez R., Palomar-Pardavé M., Romero-Romo M., Ramírez-Silva M.T. (2018). New insights on the spectrophotometric determination of melatonin pK a values and melatonin-βCD inclusion complex formation constant. Spectrochim. Acta A Mol. Biomol. Spectrosc..

[98] Wu Y. (2012). Preparation of low-molecular-weight hyaluronic acid by ozone treatment. Carbohydr. Polym..

[99] Minaberry Y., Chiappetta D.A., Sosnik A., Jobbágy M. (2013). Micro/nanostructured hyaluronic acid matrices with tuned swelling and drug release properties. Biomacromolecules.

[100] Topal B., Çetin Altindal D., Gümüsderelioglu M. (2015). Melatonin/HPβCD complex: Microwave synthesis, integration with chitosan scaffolds and inhibitory effects on MG-63CELLS. Int. J. Pharm..

[101] Yousaf A.M., Ramzan M., Shahzad Y., Mahmood T., Jamshaid M. (2018). Fabrication and in vitro characterization of fenofibric acid-loaded hyaluronic acid–polyethylene glycol polymeric composites with enhanced drug solubility and dissolution rate. Int. J. Polym. Mater. Polym. Biomater..

[102] Kader Sabbagh H.A., Hussein-Al-Ali S.H., Hussein M.Z., Abudayeh Z., Ayoub R., Abudoleh S.M. (2020). A Statistical Study on the Development of Metronidazole-Chitosan-Alginate Nanocomposite Formulation Using the Full Factorial Design. Polymers.

[103] Li Z., Kolb V.M.K., Jiang W.T., Hong H. (2010). FTIR and XRD Investigations of Tetracycline Intercalation in Smectites. Clays Clay Miner..

[104] Tran T.H., Choi J.Y., Ramasamy T., Truong D.H., Nguyen C.N., Choi H.G., Yong C.S., Kim J.O. (2014). Hyaluronic acid-coated solid lipid nanoparticles for targeted delivery of vorinostat to CD44 overexpressing cancer cells. Carbohydr. Polym..

[105] Dovedytis M., Liu Z.J., Bartlett S. (2020). Hyaluronic acid and its biomedical applications: A review. Eng. Regen..

[106] Caccavo D., Barba A.A., d’Amore M., De Piano R., Lamberti G., Rossi A., Colombo P. (2017). Modeling the modified drug release from curved shape drug delivery systems—Dome Matrix^®^. Eur. J. Pharm. Biopharm..

[107] Nechifor G., Totu E.E., Nechifor A.C., Isildak I., Oprea O., Cristache C.M. (2019). Non-resorbable nanocomposite membranes for guided bone regeneration based on polysulfone-quartz fiber grafted with nano-TiO_2_. Nanomaterials.

