# Supplementary material for: Considerations on the Controlled Delivery of Bioactive Compounds through Hyaluronic Acid Membrane
Source: Membranes (Basel). 2022 Mar 8;12(3):303. doi: 10.3390/membranes12030303 (PMC8949277; doi:10.3390/membranes12030303)
Supplement: Supplementary file 1 [file membranes-12-00303-s001.zip › membranes-1616832-supplementary.pdf]

Supplementary Material

Considerations on the controlled delivery of bioactive compounds through hydrogel membrane

Eugenia Eftimie Totu[1,*], Daniela Manuc[2,*], Tiberiu Totu[3], Corina Marilena Cristache[4], Roxana-Mădălina Buga[3], Fatih Erci[5], Camelia Cristea[6], Ibrahim Isildak[7]

Department of Analytical Chemistry and Environmental Engineering, University Politehnica of Bucharest, 1-7 Polizu St., 011061 Bucharest, Romania; eugenia.totu@upb.ro
Department of Public Health, Faculty of Dental Medicine, "Carol Davila" University of Medicine and Pharmacy, 8 Eroii Sanitari Blvd, 050474 Bucharest, Romania; daniela.manuc@umfcd.ro
School of Life Sciences, Ecole Polytechnique Fédèrale de Lausanne (EPFL), Route Cantonale, 1015 Lausanne, Switzerland; titotu@student.ethz.ch; rm.buga@yahoo.com
Department of Dental Techniques, Faculty of Midwifery and Nursing (FMAM), "Carol Davila" University of Medicine and Pharmacy, 8 Eroii Sanitari Blvd, 050474 Bucharest, Romania, corina.cristache@umfcd.ro
Department of Biotechnology, Faculty of Science, Necmettin Erbakan University, Meram-Konya, Turkey; ferci@erbakan.edu.tr
Biotechnologies Center, University of Agriculture and Veterinary Medicine, 42 Blvd. Mărăşti, 011464 Bucharest, Romania; camelia_crst@yahoo.com
Department of Bioengineering, Yildiz Technical University, Istanbul, Turkey; iisildak@yahoo.com
Correspondence: daniela.manuc@umfcd.ro, eugenia.totu@upb.ro

The present Supplementary Material comprises thermal analysis investigations, FTIR, fluorescence, and UV spectrophotometry analysis that support the main manuscript. Also, are introduced images from the microbiological assessment performed.

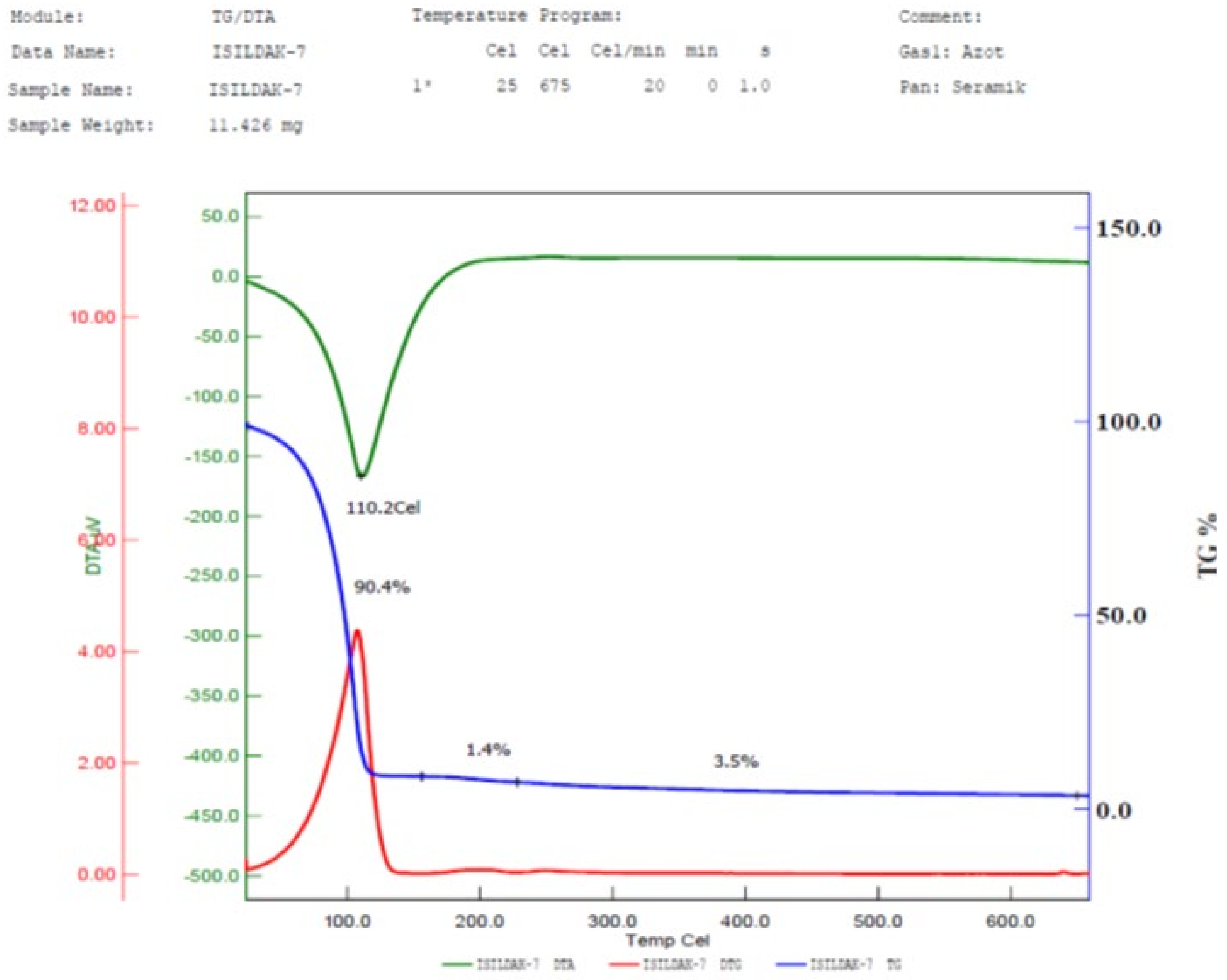

Module: TG/DTA
Data Name: ISILDAK-7
Sample Name: ISILDAK-7
Sample Weight: 11.426 mg
Temperature Program:
Cel Cel Cel/min min s
1* 25 675 20 0 1.0
Comment:
Gas1: Azot
Pan: Seramik
110.2Cel
90.4%
1.4%
3.5%
DTA uV
TG %
Temp Cel
ISILDAK-7 DTA
ISILDAK-7 DTG
ISILDAK-7 TG

a.

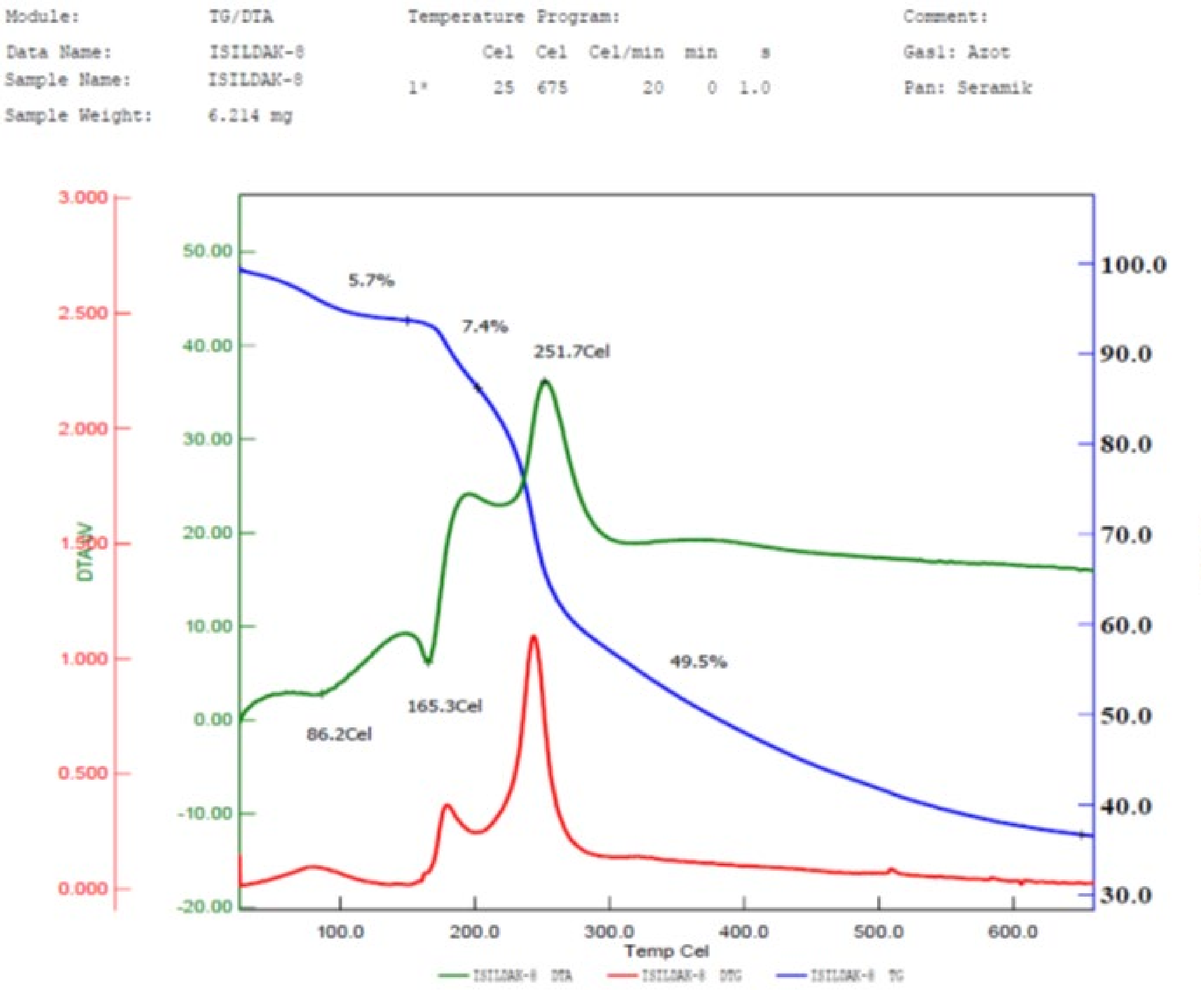

b.

Figure S1. Thermal analysis. a. HA complex matrix; b. solid complex mixture

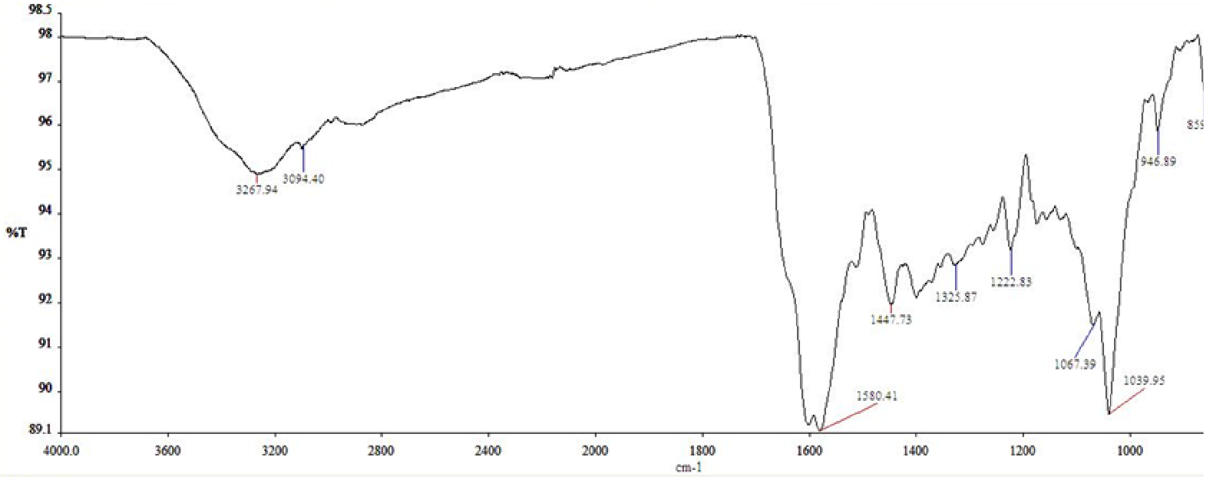

a.

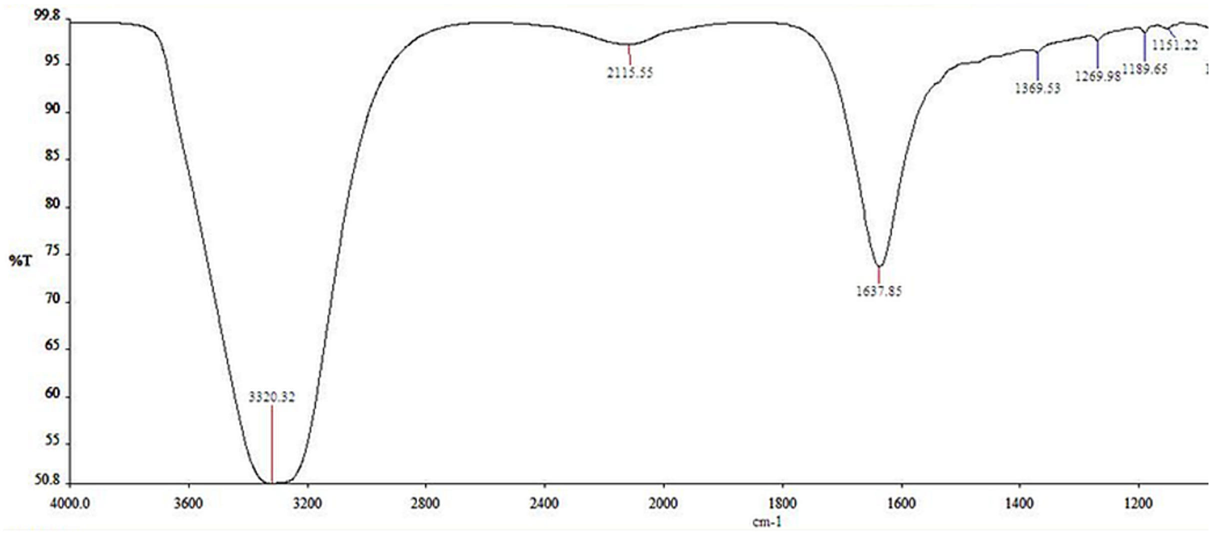

b.

Figure S2. FTIR spectra. a. solid complex mixture; b. HA matrix.

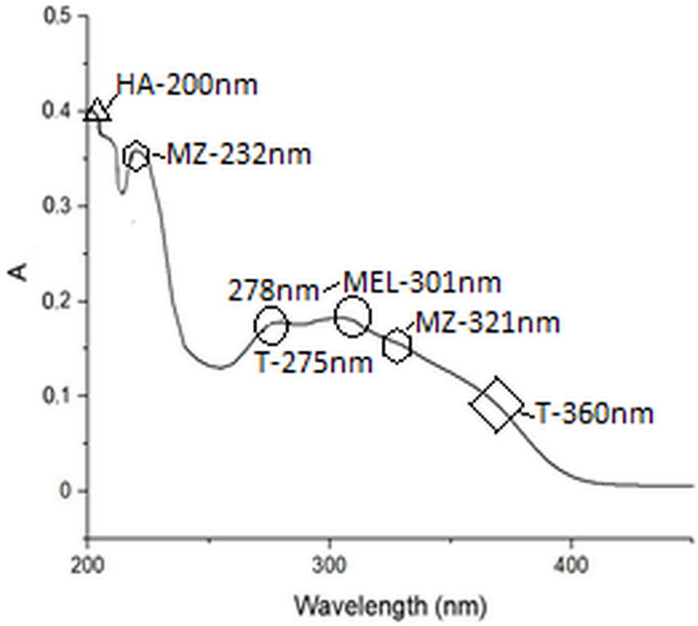

Figure S3. UV Spectrum for HA hydrogel form.

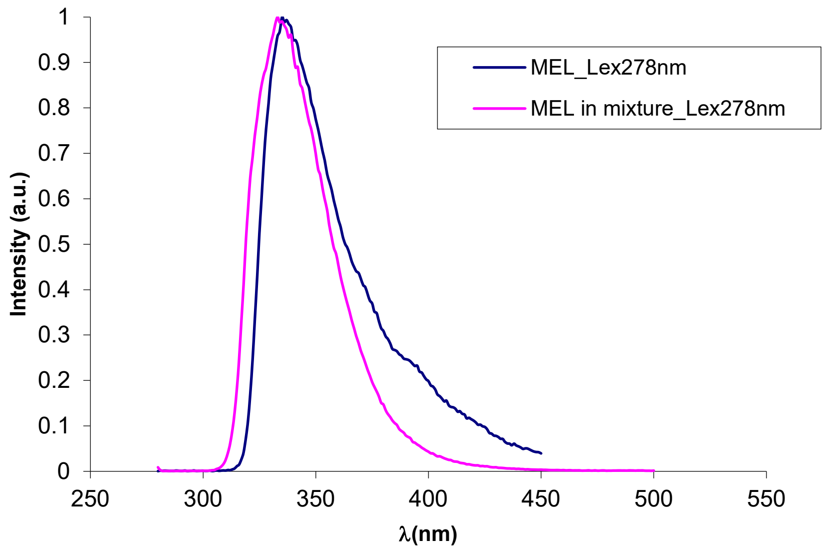

MEL_Lex278nm
MEL in mixture_Lex278nm
Intensity (a.u.)
λ(nm)
1
0.9
0.8
0.7
0.6
0.5
0.4
0.3
0.2
0.1
0
250
300
350
400
450
500
550

a.

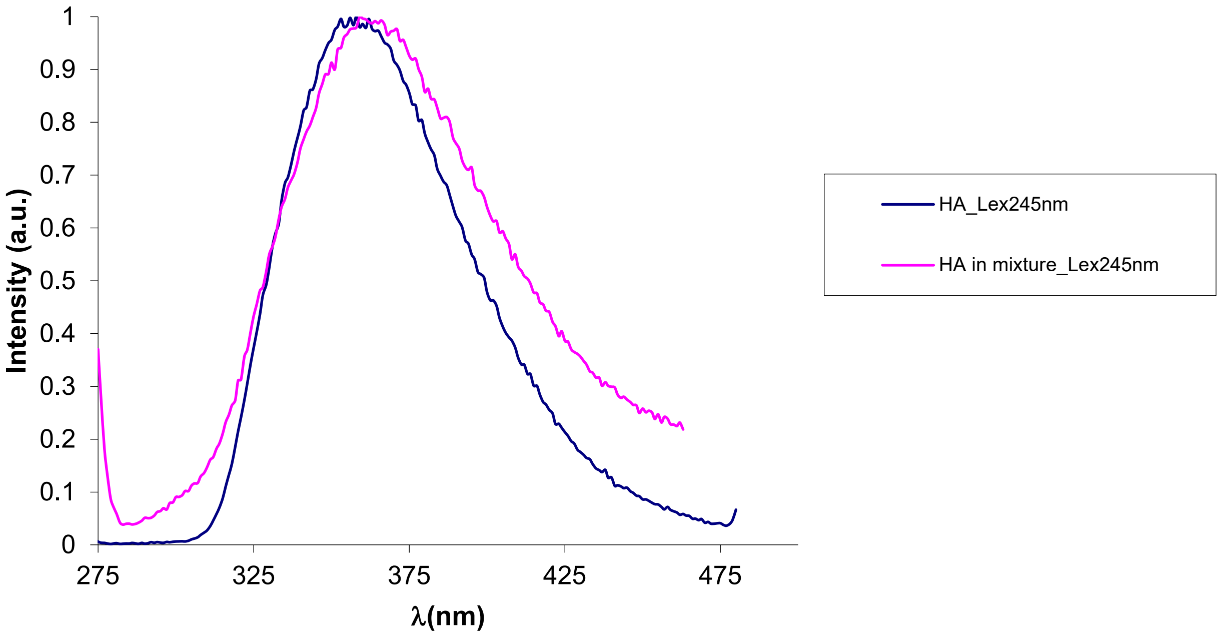

HA_Lex245nm
HA in mixture_Lex245nm
Intensity (a.u.)
λ(nm)
1
0.9
0.8
0.7
0.6
0.5
0.4
0.3
0.2
0.1
0
275
325
375
425
475

b.

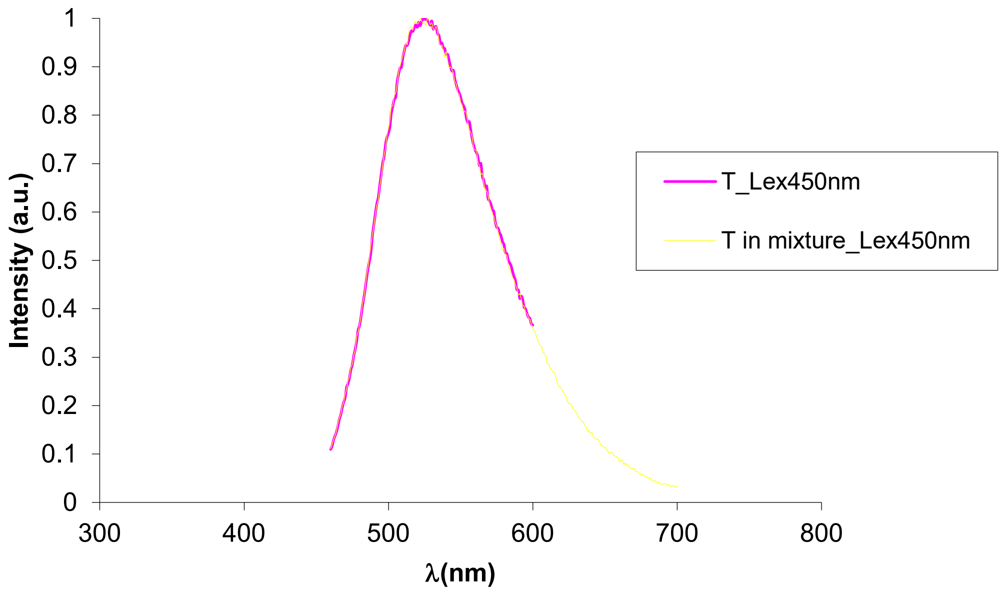

c.

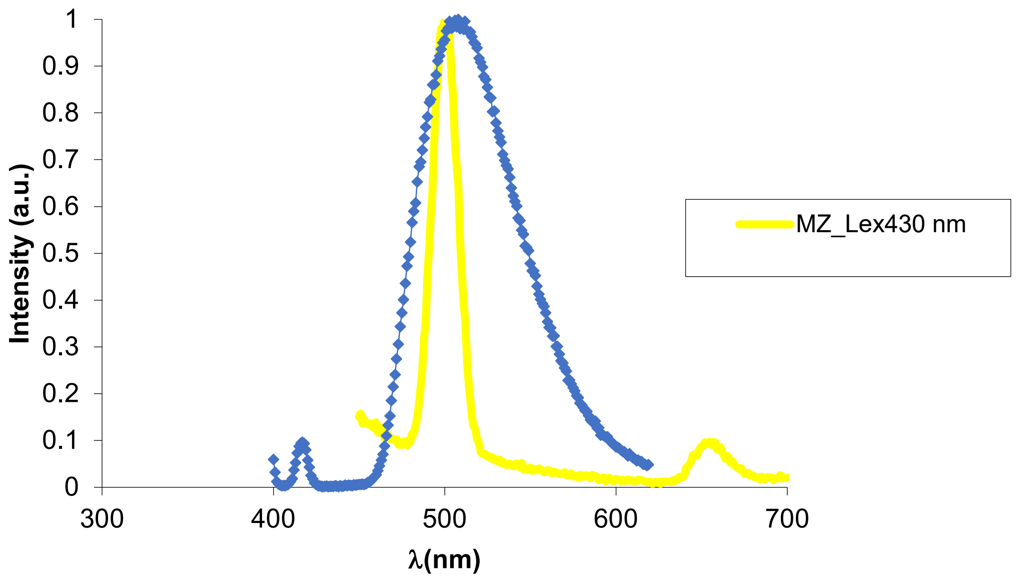

d.

Figure S4. Fluorescence spectra (detailed) for each component: individually and in complex mixture: a. melatonin-MEL; b. hyaluronic acid-HA; c. metronidazole-MZ; d. tetracycline-T.

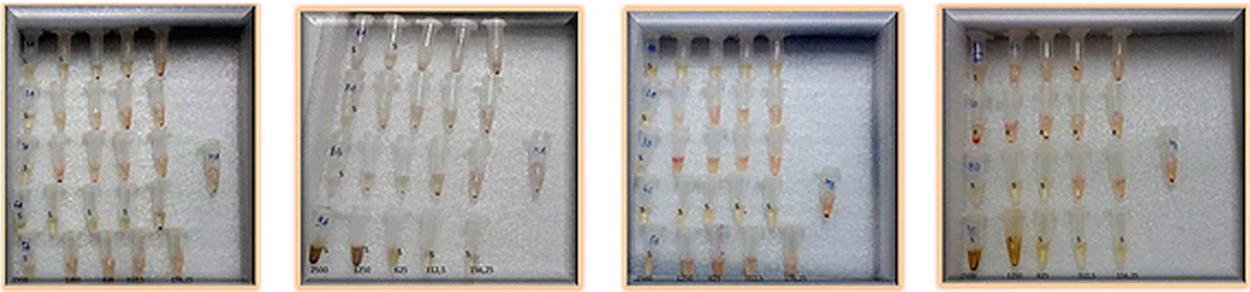

a. **b.** **c.** **d.**

Figure S5. The action of the microbial agents on the systems based on the bioactive formulation.

a. *Staphiloccocus spp*. 1-5; b. *Staphiloccocus spp*. 6-9; c. *Streptoccocus spp.* 1-5; d. *Streptoccocus spp.* 6-9.

The notations 1 to 9 correspond to 1- Tetracycline, Metronidazole, Soft paraffin; 2- Melatonin, Hyaluronic Acid, Soft paraffin; 3- Melatonin, Hyaluronic Acid – solid sample; 4- Metronidazole, Tetracycline, Melatonin, Hyaluronic Acid matrix; 5-Soft paraffin; 6- Hyaluronic Acid; 7- Melatonin; 8- Metronidazole; 9-Tetracycline.
